# Ethnic and racial differences in children and young people with respiratory and neurological post-acute sequelae of SARS-CoV-2: an electronic health record-based cohort study from the RECOVER Initiative

**DOI:** 10.1016/j.eclinm.2024.103042

**Published:** 2025-01-02

**Authors:** Suchitra Rao, Rodrigo Azuero-Dajud, Vitaly Lorman, Jeremy Landeo-Gutierrez, Kyung E. Rhee, Julie Ryu, C. Kim, Megan Carmilani, Rachel S. Gross, Sindhu Mohandas, Srinivasan Suresh, L. Charles Bailey, Victor Castro, Yalini Senathirajah, Shari Esquenazi-Karonika, Shawn Murphy, Steve Caddle, Lawrence C. Kleinman, Leah Castro-Baucom, Carlos R. Oliveira, Jonathan D. Klein, Alicia Chung, Lindsay G. Cowell, Charisse Madlock-Brown, Carol Reynolds Geary, Marion R. Sills, Lorna E. Thorpe, Jacqueline Szmuszkovicz, Kelan G. Tantisira, RECOVER EHR and, Ivan Diaz, Rachel Kenny, Parsa Mirhaji, Ravi Jhaveri, Marc Rosenman, L. Charles Bailey, Christopher Forrest, Beth Tarini, Hiroki Morizono, Nathan Pajor, W. Schuyler Jones, Kieler Curtis, Rishi Kamaleswaran, Nita Deshpande, Saul Blecker, Claudia Pulgarin, Marion Sills, Erin Hinkman, Dan Fort, Timothy Guthrie, Cynthia Chuang, Wenke Hwang, Dimitri Christakis, Daksha Ranade, Shannon Herring, Aaron Mishkin, Soledad Fernandez, Neena Thomas, Yuriy Bisyuk, Jyotsna Fuloria, Elizabeth Chrischilles, Boyd Knosp, Asa Oxner, Athanasios Tsalatsanis, Lindsay Cowell, Phillip Reeder, Stephen M. Downs, Brian Ostasiewski, Rainu Kaushal, Thomas Campion, RECOVER Pediatric Cohorts, Jessica Snowden, Katherine Irby, Paul Darden, Lexie Dixon, Danielle Evans, Connor Garbe, Laura Hobart-Porter, Lee Howard, Kathy Hummel, Hannah Krehbiel, Haley Spradlin, Phaedra Yount, Amy Elliott, Grace Adam, Jyoti Angal, Maria Barber, Katelynne Clark, Clayton Dos Reis, Mandy Freesemann, Christa Friedrich, Christine Hockett, Rachel Johannsen, Emily Johnson-Vonk, Cassidy Kaiser, Alexa Kruse, Jennifer Lang, Peter Lim, Meggie McCoy, Lorie Miller, Shelby Petereit (Cerkovnik), Jaime RiChard (Werpy), Jessica Seiler, Bret Sundleaf, Joshua Svendsen, Billy Trosper, Olivia Vermeulen, Scott Young, Aul Palumbo, Sean Dabney, Marie-Christine Fahrner, Torrey Gallagher, Karilyn Martini, Mary McNally, Sarah Vivensi Stiverson, Jessica Kosut, Venkataraman Balaraman, JoAnn Cheung, Travis K.F. Hong, Shanelle Kalua, Evan Minami, Andrea Siu, Micah Tong, Ronald J. Teufel, Andy Atz, Marina Dantas, Tyler Kasmarcak, Kreighton Milks, Judith Ross, Chijoke Ikomi, Marisa Meyer, Connie Nguyen, Gwen Pellicciotti, Thao-Ly Phan, Karen Ravin, Victoria Reynolds, Abigail Strang, Deepika Thacker, Dan Eckrich, Annabelle Goetter, Cheyenne Katz, Karen Kowal, Carol McDevitt, Emily Zimmerman, Genesis Agosto Roman, Akram Alshawabkeh, Ishwara Ayala Ortiz, Virginia Casey, Jose Cordero, Jocelyn De Jesus, Crystal Galan, Gredia Huerta-Montanez, Nilda Otero, Mayra Rivera Robles, Priscilla Roman, Genesis Roman, Zaira Rosario-Pabon, Xiodenis Santiago, Carmen Velez-Vega, Carlos Vergara, Daniel S. Hsia, Baylea Albarado, Tracey Allen, Allison Attuso, Taylor Ayers, Emily Bebler, Grace Bella, Alexa Bennett, John Brown, Alison Carville, Sydnie Darby, Kara Devall, Amber Dragg, Angela Elderedge, Elisabeth Fontenot, Greta Fry, Bethany Gildersleeve, Sara Goff, Lauren Harrington, Lisa Jones, Victoria Kaiser, Yejee Lee, Stephen Lee, Erin LeJeune, Robert Leonhard, Jennifer Levatino, Donald Lewis, Angrielle Lloyd, Ron Monce, Susannah Munro, Meghan Phillips, Blair Pucheu, Emily Rachal, Jennifer Rood, Stacey Roussel, Renee Rumsey, Connor Sanford, Monica Santos, Aryelle Stafford, Amy Thomassie, Celeste Waguespack, Katherine Walgamotte, Meredith Welch, Aubrey Windham, Michelle Stevenson, Tiffany Bell, Jackie Boyd, Soham Dasgupta, Sarah Deans, Katie Harris, Molly Hemmerle, Sarah King, Cameo McGuire, Brie Merten, Sarah Morris, Madison Ray, Brooklyn Reinhardt, Shellese Shemwell, Theresa Simeon, Katherine Walker, Sara Watson, Kathryn Weakley, Russell McCulloh, Johnathon Figliomeni, Laura Fischer, Denise Hoover, Megan Morse, Aleisha Nabower, Evan Roberts, Alice Sato, Joann Von Bon, Hengameh Raissy, David Archuleta, Rebecca Brito, Richard Campbell, Jude Chavez, Walter Dehority, Noella Garcia-Soberanez, Eve Gronert, Matthew Kadish, Jerry Larrabee, Debbie Lovato, Karen Luo, Noah Martinez, Analyse Merlino, Emily Reese, Sarah Ward, Kevin Wilson, Amanda Bogie, Ryan Brown, Ryan Butchee, Gina Fergeson, Ryan McKee, Brandon Mohler, Tiffany Moore, Valorie Owens, Sarah Stubbs, Timothy Van Wagoner, Kelly Cowan, Meghan Bethel, Laurie Chassereau, Thomas Lahiri, Lesley Cottrell, Lauren Lake, Kathy Moffett, Emily Polak, Sarah Stutler, Charlotte Workman, David Warburton, Sindhu Mohandas, John C. Wood, Emma Carpenter, Isabelle Dhindsa, Samantha Mejia, Nelly Moghadam, Candice Mulder, Sharon O'Neil, Alisha Osornio, Adrian Rios, Sydney Rosen, Andrea Smith, Deeba Tabibi, Sharon Tang, Ariana Teame, Melissa Stockwell, Joshua Milner, Erika B. Rosenzweig, Brett Anderson, Tawanda Aquino, Elizabeth Berg, Steve Caddle, Marina Catallozzi, Wendy Chung, Tom Connors, Aliva De, Anny Diaz Perez, Michael DiLorenzo, Dani Dumitriu, Kanwal Farooqi, Michael Fremed, Sylvie Goldman, Kayla Kaplan, Usha Krishnan, Aimee Layton, Angela Lignelli-Dipple, Son McClaren, Jonathan Overdevest, Michelle Rodriguez, Jay Selman, Wendy Silver, Raul Silverio, Ana Valdez de Romero, Celibell Vargas, Alan Werzberger, Daniella Caputo, Camille Leggieri, Pamela Pretsch, Lawrence Kleinman, Sunanda Gaur, Lisa Cerracchio, Amber Folnagy, Maria Gennaro, Sherri Gzemski, Yue Hao, Simon Li, Sandee Moroso, Manette Ness-Cochinwala, Akhil Patel, Benjamin Richlin, Harsh Sharma, Damaris Soto, Christian Suarez, Bibiana Vargas Forero, Lynn Olson, Kristin Davis, Alexander Fiks, Miranda Griffith, Donna Harris, Everly Macario, Jennifer Steffes, Alessandra Torres, Rangaraj Selvarangan, Dithi Banerjee, Chris Day, Kelsye Howell, Megan Mains, Juan C. Salazar, William T. Zempsky, Emily Bean, Carlie DeFelice, Hassan El Chebib, Katherine W. Herbst, Stephanie Lesmes, Ian C. Michelow, Melissa Santos, Noah Schulman, Wilson D. Pace, Alicia Brooks-Greien, Karinne Colin, Christina M. Hester, Ariadna Juarez-Colunga, Brian Manning, Joel Shields, Jack Westfall, Daphne York, Judy Aschner, Katharine Clouser, Justine Griswold, Donna Lee, Amanda Nowakowski, Maryellen Riordan, Hulya Bukulmez, David Kaelber, Rozina Aamir, Mohammed Abuzahrieh, Nandini Bangalore, Alexis Brown, Wendy Dalton, Suzanne Fortuna, Judi Minium, Bonnie Rosolowski, Sheila M. Nolan, Amal Ahmed, Suzanne Braniecki, Montserrat Contreras, Allen Dozor, Supriya Jain, Suzanne Kaseta, Sankaran Krishnan, Zachary Messer, Armando Ramirez, Aalok Singh, Randy Williams, Kyung Rhee, Kelan Tantisira, Almary Akerlundh, Natacha Akshoomoff, Maria Arroyo, Wendy Barrientos, Rakesh Bhattacharjee, Bryant Y. Chao, Maricela Diaz, Sergio Garcia, Sonia Garcia, Guadalupe Gomez, Trinidad Herrera, Margarita Holguin, Manaswitha Khare, Elizabeth Kiernan, Jeremy Landeo Gutierrez, Ileana Matta, Sofia Reyes, Julie Ryu, Cinthia Sanchez, Andrea Schreck, Megan R. Warner, Uzma Hasan, Elizabeth Ricciardi, Vanessa Trespalacios, Vince Faustino, Matthew Kluko, Carlos R. Oliveira, Kyung E. Rhee, Kelan G. Tantisira, Almary Akerlundh, Natacha Akshoomoff, Wendy Barrientos, Rakesh Bhattacharjee, Bryant Chao, Maricela Diaz, Sonia Garcia, Maria Glenn-Arroyo, Guadalupe Gomez, Trinidad Herrera, Margarita Holguin, Manaswitha Khare, Elizabeth A. Kiernan, Jeremy Landeo-Gutierrez, Anika Madan, Lisa Ramos Vallejo, Sofia Reyes, Julie Ryu, Cinthia E. Sanchez, Andrea Schreck, Maira Suarez, Megan R. Warner, Patricia Kinser, Amy Salisbury, Jocelyn Espinoza, Sara Moyer, Amy Rider, Sally Russell, Michael Schecter, Lindsey Stevenson, Cheryl R. Stein, Stephanie V. Caldas, Thomas Dylan Castro Ovalle, Anthony Chung, Jonathan S. Farkas, Maria Isidoro-Chino, Deniz Kesebir, Eugenia Kim, Ashley Quarless, Alan Schlechter, Ranjini Srinivasan, Viren D'Sa, Fatoumata Barry, Phoebe Burton, Rosa Cano Lorente, Caroline Cummins, Stephanie Wehbe, Sandra Brown, Anders Dale, Terry Jernigan, Kyung E. Rhee, Susan Tapert, Jose Aguilar, David Benjamin, Natalie C. Buchbinder, Norma Castro, Brandy Emerson, Hugh Garavan, Jennifer Graves, Amanda Guerrero, Janosch Linkersdoerfer, Robert Schooley, Wes Thompson, Thanh Trinh, Ron Yang, Megan Herting, Elizabeth Sowell, Cynthia Cisneros, Lauren Goedde, Cedric Manlhiot, Raul Gonzalez, Angela Laird, Jorge Limia, Robin Aupperle, Martin Paulus, Melanie Curry, Nour S. El-Sabbagh, Kevin Gray, Lindsay Squeglia, Samuel Agbeh, Cori Herring, Brittany Mckenzie, Bonnie Nagel, Abby Espinoza, Anthony Hill, Angie Morales, Fiona Baker, Eva Muller-Oehring, Ian Colrain, Ingrid Durley, Mirella Dapretto, Lucina Uddin, Susan Bookheimer, Christina Caldera, Jennifer Dzul, Cinthia Zarate, Marie Banich, Paola Badilla, Jennifer Keith, G. Kumar, David Messinger, Katie Prazak, Sara Jo Nixon, Melanie Cardoso, Meagan Sullivan, Stephen Villard, Linda Chang, Thomas Ernst, Christine Cloak, Huajun Liang, Meghann C. Ryan, Mary Heitzerg, Jennifer Conley, Leonard Puttler, Monica Luciana, William Iacono, Aaron Schroeder, Duncan Clark, Doyeon (Dan) Kim, Megan Retucci, Edward Freedman, John Foxe, Sile Ni Mhurchu, Samantha Spallina, Erin McGlade, Deborah Yurgelun-Todd, Liz Bell, Kirsten Cline, Lauren Heinrich, Perry Renshaw, Hugh Garavan, Alexandra Potter, Cass Barrett, Sofia Lozon, Samantha Spear, Christine Larson, Krista Lisdahl, Tory Clearwater, Christine Kaiver, Caitlin Nelson, Zach Paltzer, Bridgette Peteet, James Bjork, Mike Neale, Olive Calonge, Noah Slattery, Lisa Straub, Deanna Barch, Andrew Heath, Pamela Madden, Taylor Powell, Denise Schmitz, Dylan Gee, Boris Epie, Rebekah Hobbs, Alex Williams, Zhouran (Rick) Xiang, Julie Miller, Jane Newburger, Felicia Trachtenberg, Ayesha Amarnath, James Ambrosoli, Denise Artis, Sachin Bandari, Emily Birmingham, Lozan Eyob, Kerri Hayes, Chenwei Hu, Melissa Joyce, Valentina Kazlova, Iris Liu, Amanda Marshall, Devine Mbizdenyuy, P.J. Mu, Robin Rowe, Brooke Sayles, Mo Zhang, Pei-Ni Jone, Michael Carr, Lauren Goodell, Wantanabe Kar, Kathleen Van't Hof, Kristin Sexson, Elias Moussi, David Olukayode, Sandra Pena, Ricardo Pignatelli, Faridis Serrano, Sara Sexson Tejtel, Lara Shekerdemian, Audrey Dionne, Jane Newburger, Annette Baker, Sarah DeFerrantini, Thomas Giorgio, Numaira Khan, Simran Mahanta, MaryBeth Son, Matt Oster, Melissa Burnett, Kolby Sanders-Lewis, Suchitra Rao, Sonia Chavez, Georgia Reis, Jackie Szmuszkovicz, Fariborz Behzadian, Carla Canas, Andrew Cheng, Mike Gawad, Paige Johnson, Alicia Kazarians, Sindhu Mohandas, Anastasia Sarkissian, Brandi Scott, Consuelo Secules, Jennifer Su, Crystal Vargas, Jodie Votava-Smith, Sharon Wagner-Lees, Shuo Wang, Pierre Wong, Yamuna Sanil, Sanjeev Aggarwal, Aiman Almasnaah, Nirupama Kannikeswaran, Kathleen Meert, Gautam Singh, Priya Spencer, Nancy Sullivan, Sureja Sundaralingam, Vishnu Undyala, Emily Ward, Amanda Weber, Charmaine Williams Farr, Tamara Bradford, Marla Johnston, Matthew Elias, Alex Fiks, Chris Forrest, Dana Albizem, Susan Coffin, Therese Giglia, Katherine Lupton, Grace Marks, Tonia Morrison, Shawn O'Connor, Daniel Forsha, Jennifer Nelson, Rachel Sachdeva, Dara Watkins, Ashraf S. Harahsheh, Jordyn Britton, Alix Fetch, Anita Krishnan, Onais Tariq, Sean Lang, Marisa Almaguer, Jim Cnota, Lauryn Dugan, Elise Pickering, Kathleen Rathge, Elizabeth Mitchell, Christine Capone, Nilanjana Misra, Olga Shamailova, Mark Russell, Tammy Doman, Lori Harris, Keren Hasbani, Sagar Jani, Brian McCrindle, Jessica Bainton, Maryanne Chrisant, Doris Alaby, Norma Barton, Danielle Katz, Paulette Smith, Stephanie Handler, Joe Block, Regina Cole, Jennifer Maldonado, Kim McHugh, Andrew Atz, Megan Bickford, Jason Buckley, John Costello, Delany Dennis, Elizabeth Emrath-Zwick, Mary Freeman, Lanier Jackson, Madison Johnson, Tyler Kasmarcak, Elizabeth Mack, Scott Pletzer, Natasha Ruth, Carolyn Taylor, Sinai Zyblewski, Kanwal Farooqi, Brett Anderson, Katrina Golub, Chanel Rojas, Korsin Rosalind, Chantal Sanchez, Shubhika Srivastava, Carol Prospero, Deepika Thacker, Ed Williams, Varsha Zadokar, Arash Sabati, Kylie Domian, Ashley Herzberg, Sanjana Khanna, Todd Nowlen, Susan Park, Jade Porche, Samantha Stack, Dongngan Truong, Andrea Dunn, Lilly Fagatele, Emma Joyce, Linda Lambert, Kirsten Dummer, Sherrie Bandy, Jane Burns, Katheryn Crane, Sanjeet Hedge, Joan Pancheri, Adriana Tremoulet, Ronald Mark Payne, Mary Stumpf, Michael Portman, Hidemi Kajimoto, Camden Hebson, Krissie Hock, Onyekachukwu Osakwe, Jemylle Morato, Divya Shakti, Matt Kadish, Hengameh Raissy, Jerry Larrabee, Kavita Sharma, William Anguiano, Catherine Ikemba, Alejandra Lozano, Maria Martinez, Wendy Rojas, Lerraughn Morgan, Isaura Macias, Carl Owada, Mayra Rangel, Michelle Sykes

**Affiliations:** a Department of Pediatrics, University of Colorado School of Medicine and Children's Hospital Colorado, Aurora, CO, USA; b Applied Clinical Research Center, Children's Hospital of Philadelphia, Philadelphia, PA, USA; c Division of Respiratory Medicine, Department of Pediatrics, University of California San Diego, Rady Children's Hospital of San Diego, San Diego, CA, USA; d Department of Pediatrics, UC San Diego School of Medicine, San Diego, CA, USA; e Department of Population Health, New York University Grossman School of Medicine, New York, NY, USA; f Division of Infectious Diseases, Children's Hospital Los Angeles, Keck School of Medicine, University of Southern California, Los Angeles, CA, USA; g Divisions of Health Informatics & Emergency Medicine, Department of Pediatrics, University of Pittsburgh, UPMC Children's Hospital of Pittsburgh, Pittsburgh, PA, USA; h Research Information Science and Computing, Mass General Brigham, Somerville, MA, USA; i University of Pittsburgh School of Medicine, Pittsburgh, PA, USA; j Mass General Brigham, Harvard Medical School, Boston, MA, USA; k Columbia University Irving Medical Center, New York-Presbyterian Hospital, New York, NY, USA; l Department of Pediatrics and Child Health Institute of New Jersey Rutgers Robert Wood Johnson School of Medicine, Rutgers School of Public Health New Brunswick, NJ, USA; m Department of Pediatrics, Division of Infectious Diseases & Global Health, Yale University School of Medicine, New Haven, CT, USA; n Department of Biostatistics, Division of Health Informatics, Yale University School of Public Health, New Haven, CT, USA; o Stanford University, Palo Alto, CA, USA; p University of Illinois at Chicago/ILLInet, Chicago, IL, USA; q O'Donnell School of Public Health, UT Southwestern Medical Center, Dallas, TX, USA; r Acute and Critical Care Division, College of Nursing, University of Iowa, Iowa City, IA, USA; s University of Nebraska Medical Center, Omaha, NE, USA; t OCHIN Inc., Portland, OR, USA; u Children's Hospital of Los Angeles, Keck School of Medicine of the University of Southern California, Los Angeles, CA, USA; v University of Toronto, Toronto, Canada

**Keywords:** PEDSnet, PCORnet, Long COVID, Chronic COVID-19 syndrome, Late sequelae of COVID-19, Long-haul COVID, Long-term COVID-19, Post-COVID syndrome, Post-acute COVID-19, Post-acute sequelae of SARS-CoV-2 infection, Post-COVID condition, Ethnicity, Race, Social determinants of health

## Abstract

**Background:**

Children from racial and ethnic minority groups are at greater risk for severe acute respiratory syndrome coronavirus 2 (SARS-CoV-2) infection, but it is unclear whether they have increased risk for post-acute sequelae of SARS-CoV-2 (PASC). Our objectives were to assess whether the risk of respiratory and neurologic PASC differs by race/ethnicity and social drivers of health.

**Methods:**

We conducted a retrospective cohort study of individuals <21 years seeking care at 24 health systems across the U.S, using electronic health record (EHR) data. Our cohort included those with a positive SARS-CoV-2 molecular, serology or antigen test, or with a COVID-19, multisystem inflammatory disease in children, or PASC diagnosis from February 29, 2020 to August 1, 2022. We identified children/youth with at least 2 codes associated with respiratory and neurologic PASC. We measured associations between sociodemographic and clinical characteristics and respiratory and neurologic PASC using odds ratios and 95% confidence intervals estimated from multivariable logistic regression models adjusted for other sociodemographic characteristics, social vulnerability index or area deprivation index, time period of cohort entry, presence and complexity of chronic respiratory (respectively, neurologic) condition and healthcare utilization.

**Findings:**

Among 771,725 children in the cohort, 203,365 (26.3%) had SARS-CoV-2 infection. Among children with documented infection, 3217 children had respiratory PASC and 2009 children/youth had neurologic PASC. In logistic regression models, children <5 years (Odds Ratio [OR] 1.78, 95% CI 1.62–1.97), and of Hispanic White descent (OR 1.19, 95% CI 1.05–1.35) had higher odds of having respiratory PASC. Children/youth living in regions with higher area deprivation indices (OR 1.25, 95% CI 1.10–1.420 for 60–79th percentile) and with chronic complex respiratory conditions (OR 3.28, 95% CI 2.91–3.70) also had higher odds of respiratory PASC. In contrast, older (OR 1.57, 95% CI 1.40–1.77 for those aged 12–17 years), non-Hispanic White individuals and those with chronic pre-existing neurologic conditions (OR 2.04, 95% CI 1.78–2.35) were more likely to have a neurologic PASC diagnosis.

**Interpretation:**

Racial and ethnic differences in healthcare utilization for neurologic and respiratory PASC may reflect social drivers of health and inequities in access to care.

**Funding:**

10.13039/100000002National Institutes of Health.

**Research in context**

**Evidence before this study**

We conducted a PubMed search of the literature for articles published between March 2020 through December 2023 in English language, at the time of the compilation of the first draft of this manuscript. We first looked at the pediatric MIS-C literature using the MESH term: “Multisystem Inflammatory Syndrome in Children” as well as “Pediatric Inflammatory Multisystem Syndrome”, noting that multiple studies have shown increased rates of multi-system inflammatory syndrome in children (MIS-C) in Black and Hispanic individuals. We then used the MESH terms “Long-COVID” OR “Post-Acute Sequelae” with “Children OR Pediatric” and “Race OR Ethnicity”. Excluding reviews and editorials, we identified 17 potentially relevant retrospective and prospective cohort studies. Most of these did not specifically deal with pediatric Long-COVID, did not analyze the effect of race or ethnicity, or were limited to one specific racial or ethnic group. Of the four studies that did evaluate these factors, three demonstrated some effect of race or ethnicity on Long-COVID incidence and one small study reported no effect. No study to date has investigated subtypes of Long-COVID by race/ethnicity. Given this background, we investigated our large EHR cohort for race/ethnicity effects on Long-COVID subtypes.

**Added value of this study**

This study provides an evaluation of ethnic, racial and social factors impacting PASC in children and healthcare utilization. It highlights significant differences in these factors among children presenting with pulmonary and neurologic PASC, which may reflect social drivers of health and inequities in access to care. It also emphasizes that Long-COVID is likely a syndrome, with specific subtypes and that each of these subtypes may be associated with distinct risk profiles.

**Implications of all the available evidence**

Our findings have important public health implications regarding interventions to specific groups to decrease the risk of COVID acquisition including vaccination, or seeking treatment once infected, to mitigate the development of PASC. Our findings also impact Long-COVID research in that, by identifying different risk factors for respiratory vs. neurologic subtypes of Long-COVID, it suggests that different mechanisms likely underlie different subtypes of Long-COVID.

## Introduction

COVID-19 disproportionately affects racial and ethnic minority groups, with Hispanic and Black individuals at higher risk of infection, hospitalization, and death.^1^, ^2^, ^3^, ^4^ Emerging evidence from adult studies suggests that these groups may also be at greater risk for long COVID or post-acute sequelae of SARS-CoV-2 (PASC).^5^, ^6^, ^7^

Multiple studies have shown increased rates of multi-system inflammatory syndrome in children (MIS-C) in Black and Hispanic individuals^8^; evidence regarding racial and ethnic risk factors for other PASC manifestations in children is less established. Further, PASC encompasses a heterogeneous collection of diagnoses and affects various organ systems; therefore, it is important to assess whether the risk of PASC subtypes varies by race/ethnicity and other social determinants.

Respiratory and neurologic symptoms and conditions are some of the most common manifestations of PASC in children, including chronic cough, shortness of breath, fatigue and headache.^9^, ^10^, ^11^ Our prior work suggests that the prevalence of PASC is higher among Hispanic children.^12^ However, further examination of this finding is warranted. Exploring these associations is interwoven with social drivers of health (SDOH), which are the conditions in the environment that affect health, functioning and quality of life,^13^ including socioeconomic status, access to care and education level. Adverse SDOH may impact minority groups to a larger extent, and one must take these factors into account when evaluating racial disparities. The objectives of our study were to explore whether there were differences in the risk of respiratory and neurologic PASC by different racial and ethnic groups and SDOH using a multi-site network across the US.

## Methods

### Data source

We conducted this retrospective cohort study as part of the National Institutes of Health (NIH) Researching COVID to Enhance Recovery (RECOVER) Initiative, which seeks to understand, treat, and prevent the post-acute sequelae of SARS-CoV-2 infection (PASC). For more information on RECOVER, visit https://recovercovid.org/. We used electronic health record (EHR) data from 24 pediatric sites contributing to the US-based Patient-Centered Clinical Research Networks (PCORnet) with available geocoding data in order to ascertain social vulnerability and area deprivation indices.^14^ PCORnet is a national network of participating health systems across the US with EHR data standardized by eight large clinical research networks.^15^ Institutional Review Board (IRB) approval was obtained under Biomedical Research Alliance of New York (BRANY) protocol #21–08-508. As part of the BRANY IRB process, the protocol has been reviewed in accordance with institutional guidelines. BRANY waived the need for consent and HIPAA authorization.

We used EHR data from all healthcare encounters in outpatient, inpatient, and emergency department settings from the included health systems. Data were extracted from the RECOVER/PCORnet Database-Version s9 and included EHR data with dates of services up to July 1, 2023. The Children's Hospital of Philadelphia's Institutional Review Board designated this study as not human subjects' research and waived informed consent. Reporting of study design and results follows the reporting of studies conducted using observational routinely collected data (RECORD) guideline for observational research.^16^

### Cohorts

Our cohort included children/youth with a positive SARS-CoV-2 viral (PCR and antigen) or serology test, or who had a COVID-19/SARS-CoV-2, MIS-C or PASC diagnosis (case group) from February 29, 2020 to August 1, 2022. The cohort entry date was defined as the date of the earliest SARS-CoV-2 positive viral test or SARS-CoV-2/COVID-19 diagnosis. For individuals diagnosed with PASC without evidence of prior testing, or whose earliest evidence of COVID-19 was a positive serology test, we imputed their cohort entrance date by selection of a random date in the 28–90 days prior to their earliest PASC diagnosis or positive serology test, respectively, based on our PASC definition, and that most PASC symptoms in children develop in the 1–3 months after infection.^17^ For those diagnosed with MIS-C and no prior testing, we imputed their cohort entrance date by picking a random date in the 14–42 days prior to their earliest MIS-C diagnosis.^18^ The comparison group comprised children/youth with negative SARS-CoV-2 testing and no documentation of PASC, MIS-C, or SARS-CoV-2. The cohort entrance (index) date for the SARS-CoV-2 negative group was defined as the date of the negative viral test (a random test date was chosen if a patient had more than one negative test). A second comparison group was derived from this original comparison group comprising children/youth with evidence of a non-COVID respiratory infection (i.e., testing negative for SARS-CoV-2, no evidence of a SARS-CoV-2, MIS-C, or PASC diagnosis, with diagnosis of other respiratory virus or illness). In order for a patient to be included in the SARS-CoV-2 negative respiratory infection group, they required an occurrence of a respiratory condition that occurred within a 14-day window from the time of the negative index event date. Respiratory conditions included non-COVID conditions such as influenza, bronchiolitis, bronchitis, pneumonia, and other respiratory illnesses (Supplemental Table S1).

Inclusion criteria included children, adolescents and young adults aged <21 years, who had at least 1 health care visit in the prior 12 months (to capture an active patient population), and at least 1 healthcare visit within 180 days after the index date. We excluded children for whom geocoding data was not available. The follow-up period to identify study outcomes spanned 28 days–180 days from the cohort entry date.

### Outcomes, covariates and other variables of interest

We defined PASC based on the presence of an MIS-C (U07.1), PASC (U09.9), or “sequelae of other specified infectious and parasitic diseases” (B94.8) ICD-10-CM code on 1 or more separate visits or a COVID-19 ICD-10-CM diagnosis code or positive SARS-CoV-2 test in conjunction with a post-acute condition known to be associated with PASC from our prior work.^12^^,^^19^ Respiratory and neurologic PASC was identified based on the presence of at least two diagnostic codes known to be associated with each PASC subtype; the two diagnoses were required to be observed at least 28 days apart during the follow-up period. For example, children/youth with cough and dyspnea were considered to have the respiratory subtype and those with headache, malaise/fatigue, and cognitive dysfunction were considered to have the neurologic subtype (Supplemental Table S2). We did not conduct specific evaluations on children with both respiratory and neurologic PASC given that this was a rarer outcome.

Covariates included age (categorical variable), sex, race, ethnicity, time period of cohort entry date (quarter), acute SARS-CoV-2 severity,^20^ presence and complexity of pre-existing respiratory or neurologic chronic conditions, area deprivation index, site/region, and healthcare utilization. We defined respiratory and neurologic chronic conditions using the Pediatric Medical Complexity Algorithm (PMCA) Version 2.0^21^ which categorized children/youth as having no chronic condition, non-complex chronic conditions, or complex chronic conditions in the corresponding (respiratory or neurologic) body system. We considered diagnoses up to three years before cohort entrance. The pre-existing chronic respiratory condition covariate was included in models for the respiratory outcome, and the pre-existing chronic neurologic condition covariate was included in models for the neurologic outcome.

Healthcare utilization was defined for each type of encounter (inpatient, telehealth, outpatient, etc.). The distribution of the number of each visit type per patient prior to cohort entry was analyzed and cut-off percentile points were determined to create levels of utilization for each type of visit. The cut-off points for each visit type are summarized in Supplemental Table S3. Area Deprivation Index (ADI) is another measure of social vulnerability that ranks census block groups by socioeconomic disadvantage in an area of interest and is calculated using factors such as income, education, employment, and housing quality. A block group with a ranking of 1 indicates the lowest level of “disadvantage” within the nation and an ADI with a ranking of 100 indicates the highest level of “disadvantage”.^22^ As a sensitivity analysis, we evaluated the Social Vulnerability Index (SVI), which indicates the relative vulnerability of every U.S. census tract based on 16 different social factors. SVI values range from 0 to 1, whereby values closer to 1 indicate higher vulnerability.^23^ We also explored the census-level measures which constitute SVI subthemes including socioeconomic status, household characteristics, racial and ethnic minority status and housing type/transportation. Both measures were assigned based on geocoded residential addresses. Further details regarding these definitions are provided in Supplemental Table S4.

We estimated odds ratios and their 95% CIs for PASC and PASC subtypes from multivariable logistic regression models comparing children/youth with different race and ethnicity and ADI, adjusted for age group, time period of cohort entry, presence and complexity of pre-existing chronic respiratory (respectively, neurologic) condition, and the multi-level categorical utilization variables for each visit type described above. To examine interactions in associations between race/ethnicity, ADI, and our outcomes, we conducted the same analyses stratified by ADI quintiles. Additionally, to test for the statistical significance of effect modification, we used logistic regression models on the SARS-CoV-2 positive cohort which included race, ethnicity, ADI, along with an interaction term between race and ethnicity and ADI. We conducted additional stratified analyses by age, acute COVID-19 illness severity, and underlying neurologic or respiratory conditions. In analyses stratified by acute COVID-19 illness severity, to ensure sufficiently large samples, we created two strata by grouping together patients with asymptomatic and mild presentations and patients with moderate and severe presentations, respectively, based on severity definitions we have developed previously, which are summarized in Supplemental Table S5.^20^

We repeated the multivariable logistic regression analyses on the same outcomes in our two control cohorts to examine whether associations between outcomes and covariates in our models were specific to the SARS-CoV-2 positive cohort. We additionally ran logistic regression models on two combined cohorts of (a) SARS-CoV-2 positive and negative patients and (b) SARS-CoV-2 positive and other respiratory illness patients and included a binary covariate representing presence of SARS-CoV-2 infection to detect whether SARS-CoV-2 infection modifies the effect of the associations between our outcomes and covariates. In these models, we included interactions between COVID-19 status and race and ethnicity in one model and interactions between COVID-19 status and ADI in another. In all models, we computed scaled generalized variance inflation factors (GVIFs) to control uncertainty in model estimates due to collinearity. A threshold of 10 GVIF is commonly used to identify collinearity. Beyond this threshold, collinearity may significantly distort the regression estimates, leading to unreliable results.^24^ For SDOH factors, the category with the lowest vulnerability was chosen as the reference groups. Given that all stratified and sensitivity analyses were related to the initial hypothesis exploring relationships of race and ethnicity, Bonferroni correction was not performed. Analyses were conducted using R version 4.02.

### Role of funding source

The funder had no role in the design and conduct of the study; collection, management, analysis, and interpretation of the data; preparation, review, or approval of the manuscript; and decision to submit the manuscript for publication.

## Results

After applying our exclusions (Supplemental Figure S1), among 771,725 children in the cohort, 203,365 (26.3%) were in the SARS-CoV-2 positive group. There were 72 encounters requiring date imputation. A summary of sociodemographic characteristics between those testing positive and negative for COVID-19 is shown in Table 1. Patients who tested positive were more likely to be older, Hispanic White and Black race. Distributions of medical complexity were similar across groups. There was a higher proportion of children/youth testing positive for COVID-19 from December 2021–February 2022 to June–July 2022. Most testing occurred in outpatient office settings. In comparing characteristics between our SARS-CoV-2 cohort and our second comparison group (non-COVID respiratory infection), we found similar differences, with a higher proportion of children/youth being older, diagnosed between December 2020–February 2021 and December 2021–February 2022, and were more likely be evaluated in the emergency department and outpatient settings (Supplemental Table S6). Among those with evidence of SARS-CoV-2 infection, 3211 individuals were diagnosed with respiratory PASC, 2078 children/youth were diagnosed with neurologic PASC, and 980 individuals were diagnosed with both. For logistic regression models, clinically (OR > 1.10 or < 0.9) and statistically significant results are provided with reference groups used for comparison in parentheses.

**Table 1:** Sociodemographic characteristics SARS-CoV-2 positive and SARS CoV-2 negative groups.

Characteristic	SARS-CoV-2 positive N (%)	SARS-CoV-2 negative N (%)	SMD^a^ (standard error)
	(N = 203,365)	(N = 568,360)	
Age groups (years)			0.166 (0.003)
<5	67,291 (33.09%)	225,982 (39.76%)	
12–17	59,494 (29.25%)	142,455 (25.06%)	
6–11	53,343 (26.23%)	151,737 (26.70%)	
18–21	23,237 (11.43%)	48,186 (8.48%)	
Sex			0.038 (0.003)
Female	101,289 (49.81%)	272,377 (47.92%)	
Male	102,028 (50.17%)	295,878 (52.06%)	
Other/unknown/ambiguous	48 (0.02%)	105 (0.02%)	
Race ethnicity^b^			0.108 (0.003)
Hispanic Non-White	3999 (1.97%)	11,090 (1.95%)	
Hispanic white	25,866 (12.72%)	57,488 (10.11%)	
Non-Hispanic Asian	7985 (3.93%)	23,871 (4.20%)	
Non-Hispanic black	30,853 (15.17%)	77,549 (13.64%)	
Non-Hispanic multiple races	4085 (2.01%)	15,320 (2.70%)	
Non-Hispanic other	290 (0.14%)	1077 (0.19%)	
Non-Hispanic white	84,201 (41.40%)	249,782 (43.95%)	
Other/unknown	46,086 (22.66%)	132,183 (23.26%)	
Index event period			0.655 (0.003)
March–May 2020	2123 (1.04%)	11,478 (2.02%)	
June–August 2020	6608 (3.25%)	46,476 (8.18%)	
September–November 2020	11,207 (5.51%)	60,272 (10.60%)	
December–February 2021	23,689 (11.65%)	61,202 (10.77%)	
March–May 2021	12,235 (6.02%)	60,020 (10.56%)	
June–August 2021	12,915 (6.35%)	60,074 (10.57%)	
September–November 2021	23,033 (11.33%)	98,176 (17.27%)	
December–February 2022	75,366 (37.06%)	78,513 (13.81%)	
March–May 2022	17,384 (8.55%)	60,501 (10.64%)	
June–July 2022	18,805 (9.25%)	31,648 (5.57%)	
SARS-CoV-2 infection severity			3.302 (0.004)
Asymptomatic	31,518 (15.50%)	568,360 (100.00%)	
Mild	101,575 (49.95%)	n/a	
Moderate	53,647 (26.38%)	n/a	
Severe	16,625 (8.17%)	n/a	
Location of index visit			0.483 (0.003)
ED	28,258 (13.90%)	76,456 (13.45%)	
Inpatient	4406 (2.17%)	32,605 (5.74%)	
Other/unknown^c^	27,210 (13.38%)	45,880 (8.07%)	
Outpatient office	75,931 (37.34%)	246,811 (43.43%)	
Outpatient: test only	42,760 (21.03%)	153,926 (27.08%)	
Telehealth	24,800 (12.19%)	12,682 (2.23%)	
Degree of chronicity (PMCA)			0.046 (0.003)
Non-chronic	180,432 (88.72%)	499,834 (87.94%)	
Chronic	10,776 (5.30%)	28,340 (4.99%)	
Complex chronic	12,157 (5.98%)	40,186 (7.07%)	
Patients with chronic respiratory/pulmonary conditions			0.037 (0.003)
Non-chronic	193,573 (95.19%)	543,362 (95.60%)	
Chronic	5265 (2.59%)	11,648 (2.05%)	
Complex chronic	4527 (2.23%)	13,350 (2.35%)	
Patients with chronic neurological conditions			0.049 (0.003)
Non-chronic	196,150 (96.45%)	542,882 (95.52%)	
Chronic	1719 (0.85%)	5465 (0.96%)	
Complex chronic	5496 (2.70%)	20,013 (3.52%)	
ADI^d^ national rank			0.051 (0.003)
0–19th percentile	34,387 (16.91%)	100,551 (17.69%)	
20–39th percentile	57,849 (28.45%)	170,819 (30.05%)	
40–59th percentile	43,325 (21.30%)	118,837 (20.91%)	
60–79th percentile	35,743 (17.58%)	95,109 (16.73%)	
80–100th percentile	32,061 (15.77%)	83,042 (14.61%)	
Missing or suppressed ADI value	0 (0.00%)	2 (0.00%)	
Social vulnerability index			0.041 (0.003)
0–25th percentile	57,063 (28.06%)	167,373 (29.45%)	
25–50th percentile	43,413 (21.35%)	124,064 (21.83%)	
50–75th percentile	41,793 (20.55%)	115,041 (20.24%)	
75–100th percentile	60,968 (29.98%)	161,486 (28.41%)	
Missing or suppressed ADI value	128 (0.06%)	396 (0.07%)	
PASC	10,969 (5.39%)	n/a	0.338 (0.003)
Patients with neurological presentation	2078 (1.02%)	5, 792 (1.02%)	0.005 (0.003)
Patients with respiratory presentation	3211 (1.58%)	6373 (1.12%)	0.047 (0.003)

a SMD-standardized mean difference Given the decreased reliability of p values to compare groups with large cohort sizes, the standardized mean difference is used in studies with large cohort sizes. This summary statistic is based on the difference in mean outcome between groups divided by the outcome's standard deviation. The SMD magnitude may be interpreted using Cohen's recommendations of small (0.2), medium (0.5) and large (0.8) effect sizes.^25^

b Race/ethnicity-The cohort was allocated into Hispanic or non-Hispanic, and then non-Hispanic individuals were further categorized into the following racial groups: white, black, Asian, Multiple Race, or other. Any individual who did not have any of the aforementioned categories were categorized into other/unknown.

c Other-refers to visit types that were not categorized as inpatient, outpatient ED, telehealth or ED, and could include long term care visit, non-acute institutional, administrative visits.

d ADI-Area Deprivation Index (ADI) is a measure of social vulnerability that ranks census block groups by socioeconomic disadvantage in an area of interest and is calculated using factors such as income, education, employment, and housing quality. A block group with a ranking of 1 indicates the lowest level of “disadvantage” within the nation and an ADI with a ranking of 100 indicates the highest level of “disadvantage”.

**Fig. 1:**
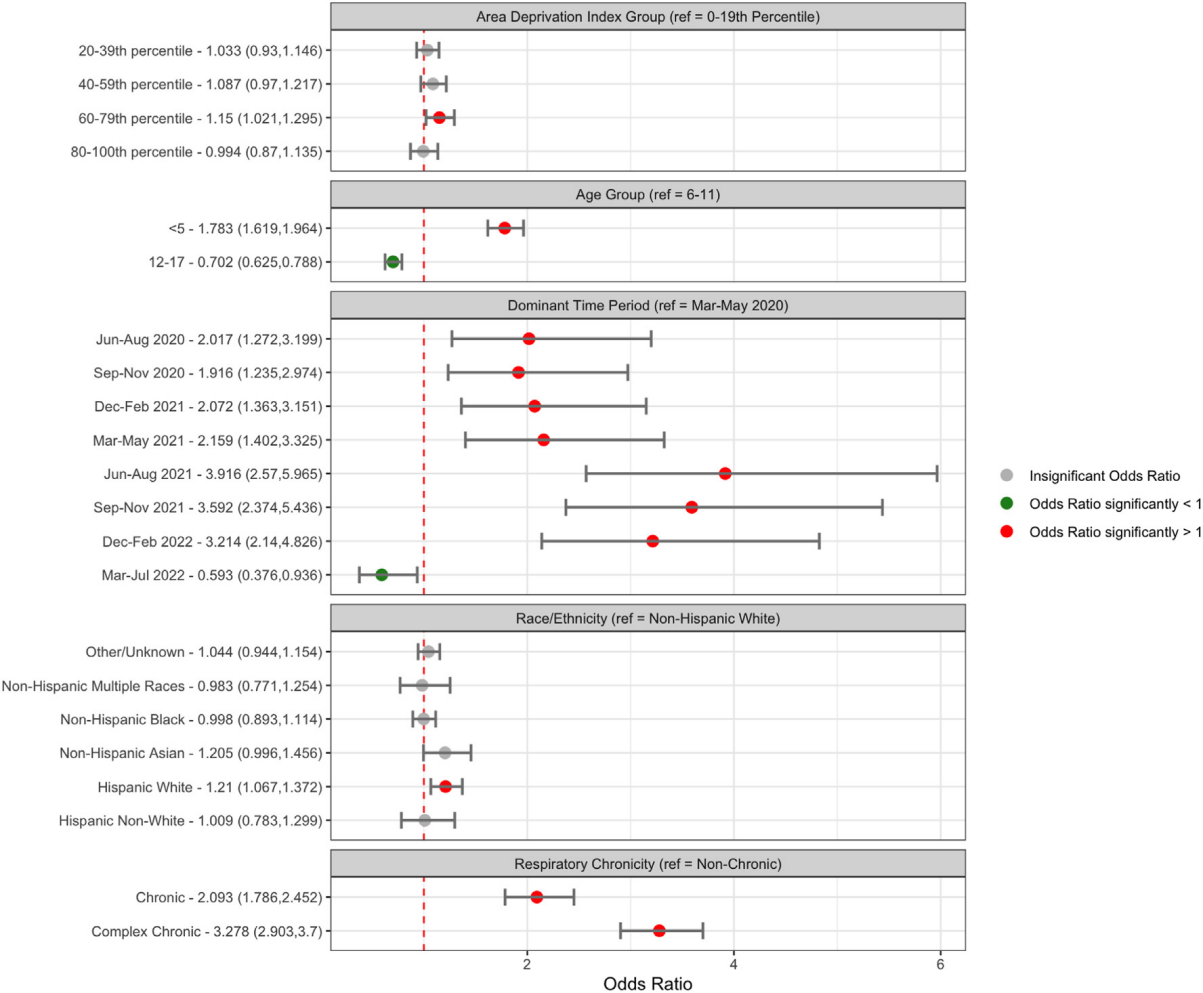
Forest plot of odds ratios (box) and 95% confidence intervals (lines) from logistic regression models evaluating the covariates associated with respiratory PASC. Models were adjusted for age group, site, race, ethnicity, area deprivation index, time period of cohort entry, presence and complexity of pre-existing chronic respiratory condition, and healthcare utilization.

In logistic regression models of children/youth with evidence of SARS-CoV-2 infection, children aged less than 5 years, Hispanic children, those with pre-existing respiratory conditions and those with higher area deprivation indices had higher risk of developing respiratory PASC. Children under 5 years of age had 1.783 times higher odds of a respiratory PASC diagnosis (95% CI 1.62–1.96) (than those aged 6–11), and those of Hispanic White race/ethnicity had 1.21 (95% CI 1.07–1.37) times higher odds of having a respiratory PASC diagnosis (than non-Hispanic White children/adolescents). When excluding asthma from the definition of respiratory PASC, there was also an increased association among those with Hispanic White background (OR 1.12, 95% CI 1.01–1.42). Children/youth living in regions with area deprivation indices in the 60th to 79th percentiles had higher odds of respiratory PASC diagnoses (OR 1.15, 95% CI 1.02–1.30) (than those in 0–19th percentiles). Individuals with pre-existing chronic complex respiratory conditions had higher odds of respiratory PASC diagnoses (OR 3.28, 95% CI 2.90–3.70) (than those with non-chronic conditions). The time period associated with the highest risk for respiratory PASC was June–November 2021 (compared with March–May 2020) (Fig. 1). The increased risk for respiratory PASC among children <5 years was also observed in the SARS-CoV-2 negative cohort, as well as the SARS-CoV-2 negative acute respiratory illness cohort. The association between Hispanic individuals and respiratory manifestations was not observed in the SARS CoV-2 negative nor SARS-CoV-2 acute respiratory illness cohorts (Supplemental Table S7). When excluding asthma from the definition of respiratory PASC, there was still an increased association among those with Hispanic White background (OR 1.12, 95% CI 1.01–1.42) (Supplemental Table S8). Hispanic youth with severe acute SARS-CoV-2 illness had higher risk of developing respiratory PASC. In analyses stratified by SARS-CoV-2 acute illness severity, the strength of the association for Hispanic ethnicity (OR 1.34, 95% CI 1.14–1.58) was higher in children/youth with more severe illness (compared with non-Hispanic white youth) but was not statistically significant for those with milder illness (OR 1.16, 95% CI 0.95–1.41) (Supplemental Table S9). In models stratified by area deprivation index, underlying respiratory condition and age, a higher risk of respiratory PASC was observed for Hispanic White individuals in the 20–39th ADI percentile (OR 1.32, 95% CI 1.05–1.67) (Supplemental Table S10), children with no pre-existing chronic respiratory conditions (OR 1.19, 95% CI 1.03–1.38) and a non-complex chronic condition (OR 1.6, 95% CI 1.04–2.47) (Supplemental Table S11), children <5 years of age (OR 1.24, 95% CI 1.04, 1.47) and 6–11 years of age (OR 1.51, 95% CI 1.17, 1.94) (Supplemental Table S12). There was no significant interaction observed between race/ethnicity and area deprivation index for respiratory PASC diagnosis (Supplemental Table S13).

**Fig. 2:**
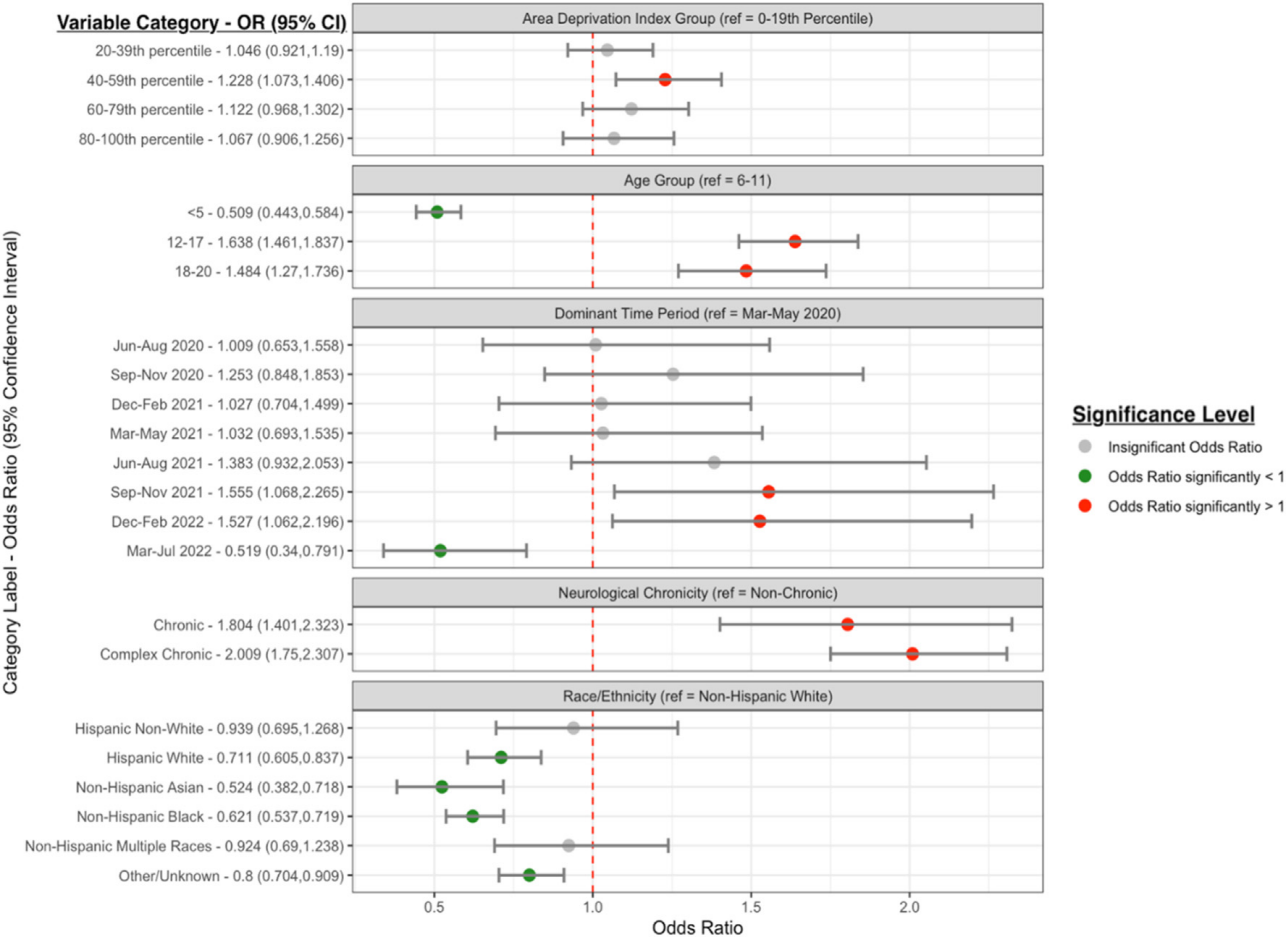
Forest plot of odds ratios (box) and 95% confidence intervals (lines) from logistic regression models evaluating the covariates associated with neurologic PASC diagnosis. Models were adjusted for age group, site, race, ethnicity, area deprivation index, time period of cohort entry, presence and complexity of pre-existing chronic neurologic condition, and healthcare utilization.

In logistic regression models of children/youth with evidence of SARS-CoV-2 infection, with similar covariate adjustments, non-Hispanic white children, older children/adolescents and those with pre-existing neurological conditions were at higher risk of developing neurologic PASC. Older children and adolescents (OR 1.48, 95% CI 1.26–1.74 for those aged 12–17 years) and those with a pre-existing neurologic condition (OR 2.187, 95% CI 1.71–2.80 for complex chronic) were more likely to have documented neurologic manifestations of PASC (compared with those aged 6–11 and those with no chronic conditions respectively). Individuals of Hispanic White race/ethnicity (OR 0.71, 95% CI 0.61–0.84) and non-Hispanic Asian (OR 0.52, 95% CI 0.38–0.72) and non-Hispanic Black race (OR 0.62, 95% CI 0.54–0.72) were less likely to have documented neurologic manifestations of PASC, compared with non-Hispanic White children. The time period with highest risk for neurologic PASC was September 2021–February 2022 (compared with March–May 2022) (Fig. 2 and Supplemental Table S14). Similar findings were observed in race/ethnicity analyses stratified by severity status for acute COVID infection and pre-existing neurologic condition, but was not significant for those with Hispanic White ethnicity in the severe group (Supplemental Table S15). Children/youth with a pre-existing, complex chronic neurologic condition were more likely to have a neurologic PASC diagnosis (OR 2.01, 95% CI 1.75–2.31) (compared with those with non-chronic conditions), but this increased risk by medical complexity was not observed for a specific racial/ethnic group in stratified analyses (Supplemental Table S16). We did not observe excessive multicollinearity for any covariate in any of the non-interaction models; all scaled GVIFs were less than 1.2; well below thresholds of concern (which range from 3 to 10 in the literature).

Some area level deprivation indices were associated with increased risk of respiratory and neurologic PASC. In secondary analyses focusing on the role of social vulnerability in PASC, we replaced ADI with 16 census-level variables which comprise SVI, and found significant increased odds for respiratory PASC diagnoses in children and youth living in regions with highest vulnerability percentiles (60th–99th), for no high school diploma, and the 40th–60th percentile for no health insurance. The significant factors increasing the risk for neurologic PASC diagnoses included living in regions with <150% poverty levels, highest vulnerability indices for crowding and no high school diploma (Supplemental Figures S2 and S3).

## Discussion

In our retrospective cohort study evaluating children, adolescents, and young adults with respiratory and neurologic PASC diagnoses using EHR data from PCORnet sites, we found that while those of Hispanic ethnicity and Black race were more likely to be diagnosed with SARS-CoV-2 infection, both groups were not at higher risk of neurologic PASC, and children and youth of Black race were not at higher risk of respiratory or neurologic PASC. Children/youth of Hispanic ethnicity, those younger than 5 years of age, with underlying respiratory conditions or with higher levels of socioeconomic disadvantage were more likely to be diagnosed with respiratory PASC. In contrast, non-Hispanic white children/youth and those 10 years of age and older were more likely to be diagnosed with neurologic PASC. The association was stronger among those with higher levels of ADI than those with lower levels of ADI. This study evaluates important sociodemographic and social vulnerability characteristics among specific subtypes of diagnosed PASC highlights the importance of assessing and addressing the social needs and health disparities of individuals with long COVID.

Our prior work demonstrated an increased risk of COVID-19 and PASC among children with Black race and Hispanic ethnicity,^12^ similar to US Census Bureau data.^26^ However, our current findings suggest an increase in respiratory PASC diagnoses in Hispanic children, but racial and ethnic minority groups were not found to be at higher risk for neurologic PASC diagnoses. Indeed, Hispanic Whites, Asians, and Blacks had lower odds of this outcome. The reasons for these differences likely relate to the complex interplay between SDOH,^27^ life stressors,^28^ healthcare seeking behaviors and access to care.^29^ which outweigh other clinical factors including underlying susceptibility due to increased comorbidity risks,^30^ genetic factors or differences in vaccination rates.^31^ Caring for children and youth with PASC increases healthcare utilization and places financial burdens on families, as well as the healthcare system. Minority groups representing socially vulnerable populations may not seek advanced care for their children for chronic issues such as headaches, concentration difficulties, or fatigue. Certain minority groups are more likely to engage in front line occupations, and thus are at increased risk of contracting and transmitting SARS-CoV-2. This limited use of healthcare may be due to factors such as decreased ability to miss work/school, healthcare costs, and lack of health insurance. Minority groups also state feeling stigmatized by healthcare professionals when seeking care for these types of symptoms, as reported in a recent survey.^27^ In the context of this study, race and ethnicity are not represented as biologic variables, but more as markers of inequities that affect marginalized individuals. Of note, we identified lower odds of respiratory PASC among higher percentiles for living in group quarters and multi-unit structures and no vehicle, and among neurologic PASC for higher percentiles for English language proficiency, group quarters, housing burden and no vehicle. These findings require further exploration, and may reflect decreased access to care rather than being true protective factors. Some of our pediatric findings overlap with those reported in the corresponding adult EHR RECOVER cohort studies from PCORnet institutions. Hospitalized adult Hispanic patients diagnosed with COVID-19 had higher odds of dyspnea compared with hospitalized adult White patients. However, in contrast to our study, Black and Hispanic adult patients had higher odds of documented headaches compared with White patients.^7^ In another study of adult Long COVID patients, Black race, mixed ethnicity, and other minority groups associated with a higher risk reporting of Long COVID symptoms compared to White ethnic groups. This risk had an inverse relationship with the level of socioeconomic deprivation, where the most socioeconomically deprived patients experienced an 11% increased risk of reporting Long COVID symptoms.^5^ However, other studies indicate lower odds of post-COVID conditions among those with black race.^32^

Strengths of this study include the large scale, diverse geographic representation, different types of medical encounters, patient identification using a combination of test results and codes, and the inclusion of control groups. We included a large representative sample of children and youth from diverse geographical locations across the U.S., including data from outpatient, ED, and inpatient encounters. Our cohort is comprised of individuals with positive SARS-CoV-2 test results as well as COVID- and PASC-related codes, enhancing data capture and accuracy. Given the unreliability of Long-COVID codes in the EHR, particularly in children, which may bias our findings, we used a comprehensive list of relevant SNOMED codes reviewed by subject matter and patient and parent advocates, which has been validated using chart reviews and analytic approaches, and further restricted the cohort to those with respiratory and neurologic manifestations. We conducted extensive sensitivity analyses including different numbers of encounters used to classify patients with PASC diagnoses, and different evaluation periods, with similar findings. Further validating our work, we analyzed findings using a SARS-CoV-2-negative cohort and a SARS-CoV-2-negative cohort with acute respiratory illnesses cohort and did not find the same race/ethnicity associations.

Our findings are subject to several limitations inherent to studies using EHR data. First, we identified symptoms and conditions that were significant enough to prompt a health care visit and be coded as a reason for an encounter, and our stringent definition likely underrepresents the true symptom burden experienced by youth with neurologic and respiratory PASC. Next, there are currently no standard definitions of respiratory or neurologic PASC, and our definitions were determined by our prior work, literature review and expert opinion. Thus comparisons to other long COVID studies remain challenging, and is a topic which requires prioritization from the long COVID scientific community. Further, sites captured in our study with sufficient data quality and geocoding data may skew our cohort to larger, academic institutions. There are some PASC conditions for which ICD-10 codes do not exist, such as brain fog, or that may not be appropriately coded. Children, adolescents and young adults with neurologic PASC may not present to care until many months after SARS-CoV-2 infection. However, we did not identify significant differences in our findings when expanding our evaluation period out to 1 year from the initial infection. We attempted to adjust for differences in healthcare utilization in our analyses but may not have been able to fully avoid biases introduced due to differences in healthcare seeking behaviors and access to care in this cohort. We were unable to look for associations with other important covariates such as pollution levels, vaccination status, smoke exposure, English as a primary language, or other factors which may influence health seeking behaviors. There is a risk of misclassification bias from incomplete capture of data during the Omicron variant period, during which home antigen testing became more widespread. Misclassification of patients with de novo conditions may have occurred, in which they were categorized as having PASC, but the condition may have been unrelated to COVID-19 infection. Finally, we were unable to formally explore the complex relationships between race/ethnicity and social drivers of health, and whether biologic factors contribute to racial and ethnic differences. Future studies by our team will seek to explore this relationship through mediation analyses.

Younger, Hispanic children/youth with underlying pulmonary conditions and with higher social vulnerability were more likely to be diagnosed with pulmonary PASC, whereas older, White children were more likely to be diagnosed with neurologic PASC. Our findings have important public health implications for outreach to specific racial and ethnic groups regarding taking steps to decrease the risk of COVID acquisition including vaccination, or seeking treatment once infected, to mitigate the development of PASC.

## Contributors

**Conceptualization:** SR, KGT, JL-G, KER.

**Methodology:** SR, KGT, RAD, VL, JL-G, KER, JR, LCK, RSG, SM, SS, CB, VC, YS, CRO, MC, LCB, CK.

**Formal analysis:** RAD, VL.

**Data Curation:** RAD, VL.

**Writing–Original Draft:** SR, KGT.

**Writing–Review & Editing:** All authors.

**Project administration:** SR, SE-K.

**Funding acquisition:** SR, LCB, LET, KGT.

**Guarantors:** SR, LCB, RAD, VL accepts full responsibility for data analysis and data integrity. SR accepts full responsibility for the work and the conduct of the study, had access to the data, and controlled the decision to publish.

## Data sharing statement

Data collected for the study will be made available upon reasonable request after publication. Data will include individual de-identified participant data and the data dictionary. Requests can be addressed to the corresponding author. Requests will be examined by a committee of relevant people involved in the study. The scientific aspects of the proposal and the ethical and legal implications of data sharing will be considered. Data will be shared after approval of the proposal and after signing of a data sharing agreement by all parties involved.

## Declaration of interests

Dr. Kleinman is Board Member for Dartnet Institute, and owns stocks with Glaxo, Amgen, Regeneron and Sanofi. Dr. Oliveira is on the Board of Directors for Eastern Society of Pediatric Research (ESPR) Executive Committee for American Academy of Pediatrics–Section on Epidemiology, Public Health, and Evidence (AAP-SOEPHE) Associate Editor for Journal of Pediatric Infectious Diseases Society. All other authors have no conflicts of interest to disclose.

## Appendix A Supplementary data

RECOVER EHR Consortium Members

RECOVER Pediatric Consortium Members

Supplemental Material Appendix 1

Supplemental Material PASC Ethnicity manuscript

All_packages_used
