# Supplementary material for: Ethnic and racial differences in children and young people with respiratory and neurological post-acute sequelae of SARS-CoV-2: an electronic health record-based cohort study from the RECOVER Initiative
Source: eClinicalMedicine. 2025 Jan 2;80:103042. doi: 10.1016/j.eclinm.2024.103042 (PMC11753962; doi:10.1016/j.eclinm.2024.103042)
Supplement: RECOVER EHR Consortium Members [file mmc1.docx]

**RECOVER EHR Consortium Members**

| First Name | Surname | Affiliation |
| --- | --- | --- |
| Ivan | Diaz | NYU Langone |
| Rachel | Kenny | NYU Langone |
| Parsa | Mirhaji | Albert Einstein College of Medicine |
| Ravi | Jhaveri | Ann & Robert H. Lurie Children's Hospital of Chicago |
| Marc | Rosenman | Ann & Robert H. Lurie Children's Hospital of Chicago |
| L. Charles | Bailey | Children's Hospital of Philadelphia |
| Christopher | Forrest | Children's Hospital of Philadelphia |
| Beth | Tarini | Children's National Hospital |
| Hiroki | Morizono | Children's National Hospital |
| Nathan | Pajor | Cincinnati Children's Hospital Medical Center |
| W. Schuyler | Jones | Duke University Health System |
| Kieler | Curtis | Duke University Health System |
| Rishi | Kamaleswaran, | Emory University |
| Nita | Deshpande | Emory University |
| Saul | Blecker | New York University Langone Health |
| Claudia | Pulgarin | New York University Langone Health |
| Marion | Sills | OCHIN, Inc. |
| Erin | Hinkman | OCHIN, Inc. |
| Dan | Fort | Ochsner Health System |
| Timothy | Guthrie | Ochsner Health System |
| Cynthia | Chuang | Penn State U College of Medicine |
| Wenke | Hwang | Penn State U College of Medicine |
| Dimitri | Christakis | Seattle Children's Hospital |
| Daksha | Ranade | Seattle Children's Hospital |
| Shannon | Herring | Temple University |
| Aaron | Mishkin | Temple University |
| Soledad, | Fernandez | The Ohio State University |
| Neena | Thomas | The Ohio State University |
| Yuriy | Bisyuk | University Medical Center New Orleans |
| Jyotsna | Fuloria | University Medical Center New Orleans |
| Elizabeth | Chrischilles | University of Iowa |
| Boyd | Knosp | University of Iowa |
| Asa | Oxner | University of South Florida |
| Athanasios | Tsalatsanis | University of South Florida |
| Lindsay | Cowell | University of Texas Southwestern Medical Center |
| Phillip | Reeder | University of Texas Southwestern Medical Center |
| Stephen M | Downs | Wake Forest School of Medicine |
| Brian | Ostasiewski | Wake Forest School of Medicine |
| Rainu | Kaushal | Weill Cornell Medicine |
| Thomas | Campion | Weill Cornell Medicine |
