# Supplementary material for: Ethnic and racial differences in children and young people with respiratory and neurological post-acute sequelae of SARS-CoV-2: an electronic health record-based cohort study from the RECOVER Initiative
Source: eClinicalMedicine. 2025 Jan 2;80:103042. doi: 10.1016/j.eclinm.2024.103042 (PMC11753962; doi:10.1016/j.eclinm.2024.103042)
Supplement: RECOVER Pediatric Consortium Members [file mmc2.docx]

**RECOVER-Pediatric Consortium Members**

| **First name** | **Last name** | **Affiliation** |
| --- | --- | --- |
| Jessica | Snowden | Arkansas Children’s Research Institute |
| Katherine | Irby | Arkansas Children’s Research Institute |
| Paul | Darden | Arkansas Children’s Research Institute |
| Lexie | Dixon | Arkansas Children’s Research Institute |
| Danielle | Evans | Arkansas Children’s Research Institute |
| Connor | Garbe | Arkansas Children’s Research Institute |
| Laura | Hobart-Porter | Arkansas Children’s Research Institute |
| Lee | Howard | Arkansas Children’s Research Institute |
| Kathy | Hummel | Arkansas Children’s Research Institute |
| Hannah | Krehbiel | Arkansas Children’s Research Institute |
| Haley | Spradlin | Arkansas Children’s Research Institute |
| Phaedra | Yount | Arkansas Children’s Research Institute |
| Amy | Elliott | Avera Research Institute |
| Grace | Adam | Avera Research Institute |
| Jyoti | Angal | Avera Research Institute |
| Maria | Barber | Avera Research Institute |
| Katelynne | Clark | Avera Research Institute |
| Clayton | Dos Reis | Avera Research Institute |
| Mandy | Freesemann | Avera Research Institute |
| Christa | Friedrich | Avera Research Institute |
| Christine | Hockett | Avera Research Institute |
| Rachel | Johannsen | Avera Research Institute |
| Emily | Johnson-Vonk | Avera Research Institute |
| Cassidy | Kaiser | Avera Research Institute |
| Alexa | Kruse | Avera Research Institute |
| Jennifer | Lang | Avera Research Institute |
| Peter | Lim | Avera Research Institute |
| Meggie | McCoy | Avera Research Institute |
| Lorie | Miller | Avera Research Institute |
| Shelby | Petereit (Cerkovnik) | Avera Research Institute |
| Jaime | RiChard (Werpy) | Avera Research Institute |
| Jessica | Seiler | Avera Research Institute |
| Bret | Sundleaf | Avera Research Institute |
| Joshua | Svendsen | Avera Research Institute |
| Billy | Trosper | Avera Research Institute |
| Olivia | Vermeulen | Avera Research Institute |
| Scott | Young | Avera Research Institute |
| aul | Palumbo | Dartmouth Hitchcock Medical Center |
| Sean | Dabney | Dartmouth Hitchcock Medical Center |
| Marie-Christine | Fahrner | Dartmouth Hitchcock Medical Center |
| Torrey | Gallagher | Dartmouth Hitchcock Medical Center |
| Karilyn | Martini | Dartmouth Hitchcock Medical Center |
| Mary | McNally | Dartmouth Hitchcock Medical Center |
| Sarah | Vivensi Stiverson | Dartmouth Hitchcock Medical Center |
| Jessica | Kosut | Kapiolani Medical Center for Women and Children |
| Venkataraman | Balaraman | Kapiolani Medical Center for Women and Children |
| JoAnn | Cheung | Kapiolani Medical Center for Women and Children |
| Travis | K.F. Hong | Kapiolani Medical Center for Women and Children |
| Shanelle | Kalua | Kapiolani Medical Center for Women and Children |
| Evan | Minami | Kapiolani Medical Center for Women and Children |
| Andrea | Siu | Kapiolani Medical Center for Women and Children |
| Micah | Tong | Kapiolani Medical Center for Women and Children |
| Ronald J. | Teufel II | Medical University of South Carolina |
| Andy | Atz | Medical University of South Carolina |
| Marina | Dantas | Medical University of South Carolina |
| Tyler | Kasmarcak | Medical University of South Carolina |
| Kreighton | Milks | Medical University of South Carolina |
| Judith | Ross | Nemours Children’s Health System |
| Chijoke | Ikomi | Nemours Children’s Health System |
| Marisa | Meyer | Nemours Children’s Health System |
| Connie | Nguyen | Nemours Children’s Health System |
| Gwen | Pellicciotti | Nemours Children’s Health System |
| Thao-Ly | Phan | Nemours Children’s Health System |
| Karen | Ravin | Nemours Children’s Health System |
| Victoria | Reynolds | Nemours Children’s Health System |
| Abigail | Strang | Nemours Children’s Health System |
| Deepika | Thacker | Nemours Children’s Health System |
| Dan | Eckrich | Nemours Children’s Hospital Delaware |
| Annabelle | Goetter | Nemours Children’s Hospital Delaware |
| Cheyenne | Katz | Nemours Children’s Hospital Delaware |
| Karen | Kowal | Nemours Children’s Hospital Delaware |
| Carol | McDevitt | Nemours Children’s Hospital Delaware |
| Emily | Zimmerman | Northeastern University, Puerto Rico Testsite for Exploring Contamination Threats |
| Genesis | Agosto Roman | Northeastern University, Puerto Rico Testsite for Exploring Contamination Threats |
| Akram | Alshawabkeh | Northeastern University, Puerto Rico Testsite for Exploring Contamination Threats |
| Ishwara | Ayala Ortiz | Northeastern University, Puerto Rico Testsite for Exploring Contamination Threats |
| Virginia | Casey | Northeastern University, Puerto Rico Testsite for Exploring Contamination Threats |
| Jose | Cordero | Northeastern University, Puerto Rico Testsite for Exploring Contamination Threats |
| Jocelyn | De Jesus | Northeastern University, Puerto Rico Testsite for Exploring Contamination Threats |
| Crystal | Galan | Northeastern University, Puerto Rico Testsite for Exploring Contamination Threats |
| Gredia | Huerta-Montanez | Northeastern University, Puerto Rico Testsite for Exploring Contamination Threats |
| Nilda | Otero | Northeastern University, Puerto Rico Testsite for Exploring Contamination Threats |
| Mayra | Rivera Robles | Northeastern University, Puerto Rico Testsite for Exploring Contamination Threats |
| Priscilla | Roman | Northeastern University, Puerto Rico Testsite for Exploring Contamination Threats |
| Genesis | Roman | Northeastern University, Puerto Rico Testsite for Exploring Contamination Threats |
| Zaira | Rosario-Pabon | Northeastern University, Puerto Rico Testsite for Exploring Contamination Threats |
| Xiodenis | Santiago | Northeastern University, Puerto Rico Testsite for Exploring Contamination Threats |
| Carmen | Velez-Vega | Northeastern University, Puerto Rico Testsite for Exploring Contamination Threats |
| Carlos | Vergara | Northeastern University, Puerto Rico Testsite for Exploring Contamination Threats |
| Daniel S. | Hsia | Pennington Biomedical Research Center |
| Baylea | Albarado | Pennington Biomedical Research Center |
| Tracey | Allen | Pennington Biomedical Research Center |
| Allison | Attuso | Pennington Biomedical Research Center |
| Taylor | Ayers | Pennington Biomedical Research Center |
| Emily | Bebler | Pennington Biomedical Research Center |
| Grace | Bella | Pennington Biomedical Research Center |
| Alexa | Bennett | Pennington Biomedical Research Center |
| John | Brown | Pennington Biomedical Research Center |
| Alison | Carville | Pennington Biomedical Research Center |
| Sydnie | Darby | Pennington Biomedical Research Center |
| Kara | Devall | Pennington Biomedical Research Center |
| Amber | Dragg | Pennington Biomedical Research Center |
| Angela | Elderedge | Pennington Biomedical Research Center |
| Elisabeth | Fontenot | Pennington Biomedical Research Center |
| Greta | Fry | Pennington Biomedical Research Center |
| Bethany | Gildersleeve | Pennington Biomedical Research Center |
| Sara | Goff | Pennington Biomedical Research Center |
| Lauren | Harrington | Pennington Biomedical Research Center |
| Lisa | Jones | Pennington Biomedical Research Center |
| Victoria | Kaiser | Pennington Biomedical Research Center |
| Yejee | Lee | Pennington Biomedical Research Center |
| Stephen | Lee | Pennington Biomedical Research Center |
| Erin | LeJeune | Pennington Biomedical Research Center |
| Robert | Leonhard | Pennington Biomedical Research Center |
| Jennifer | Levatino | Pennington Biomedical Research Center |
| Donald | Lewis | Pennington Biomedical Research Center |
| Angrielle | Lloyd | Pennington Biomedical Research Center |
| Ron | Monce | Pennington Biomedical Research Center |
| Susannah | Munro | Pennington Biomedical Research Center |
| Meghan | Phillips | Pennington Biomedical Research Center |
| Blair | Pucheu | Pennington Biomedical Research Center |
| Emily | Rachal | Pennington Biomedical Research Center |
| Jennifer | Rood | Pennington Biomedical Research Center |
| Stacey | Roussel | Pennington Biomedical Research Center |
| Renee | Rumsey | Pennington Biomedical Research Center |
| Connor | Sanford | Pennington Biomedical Research Center |
| Monica | Santos | Pennington Biomedical Research Center |
| Aryelle | Stafford | Pennington Biomedical Research Center |
| Amy | Thomassie | Pennington Biomedical Research Center |
| Celeste | Waguespack | Pennington Biomedical Research Center |
| Katherine | Walgamotte | Pennington Biomedical Research Center |
| Meredith | Welch | Pennington Biomedical Research Center |
| Aubrey | Windham | Pennington Biomedical Research Center |
| Michelle | Stevenson | University of Louisville Research Foundation |
| Tiffany | Bell | University of Louisville Research Foundation |
| Jackie | Boyd | University of Louisville Research Foundation |
| Soham | Dasgupta | University of Louisville Research Foundation |
| Sarah | Deans | University of Louisville Research Foundation |
| Katie | Harris | University of Louisville Research Foundation |
| Molly | Hemmerle | University of Louisville Research Foundation |
| Sarah | King | University of Louisville Research Foundation |
| Cameo | McGuire | University of Louisville Research Foundation |
| Brie | Merten | University of Louisville Research Foundation |
| Sarah | Morris | University of Louisville Research Foundation |
| Madison | Ray | University of Louisville Research Foundation |
| Brooklyn | Reinhardt | University of Louisville Research Foundation |
| Shellese | Shemwell | University of Louisville Research Foundation |
| Theresa | Simeon | University of Louisville Research Foundation |
| Katherine | Walker | University of Louisville Research Foundation |
| Sara | Watson | University of Louisville Research Foundation |
| Kathryn | Weakley | University of Louisville Research Foundation |
| Russell | McCulloh | University of Nebraska Medical Center and Children's Nebraska |
| Johnathon | Figliomeni | University of Nebraska Medical Center and Children's Nebraska |
| Laura | Fischer | University of Nebraska Medical Center and Children's Nebraska |
| Denise | Hoover | University of Nebraska Medical Center and Children's Nebraska |
| Megan | Morse | University of Nebraska Medical Center and Children's Nebraska |
| Aleisha | Nabower | University of Nebraska Medical Center and Children's Nebraska |
| Evan | Roberts | University of Nebraska Medical Center and Children's Nebraska |
| Alice | Sato | University of Nebraska Medical Center and Children's Nebraska |
| Joann | Von Bon | University of Nebraska Medical Center and Children's Nebraska |
| Hengameh | Raissy | University of New Mexico Health Sciences Center |
| David | Archuleta | University of New Mexico Health Sciences Center |
| Rebecca | Brito | University of New Mexico Health Sciences Center |
| Richard | Campbell | University of New Mexico Health Sciences Center |
| Jude | Chavez | University of New Mexico Health Sciences Center |
| Walter | Dehority | University of New Mexico Health Sciences Center |
| Noella | Garcia-Soberanez | University of New Mexico Health Sciences Center |
| Eve | Gronert | University of New Mexico Health Sciences Center |
| Matthew | Kadish | University of New Mexico Health Sciences Center |
| Jerry | Larrabee | University of New Mexico Health Sciences Center |
| Debbie | Lovato | University of New Mexico Health Sciences Center |
| Karen | Luo | University of New Mexico Health Sciences Center |
| Noah | Martinez | University of New Mexico Health Sciences Center |
| Analyse | Merlino | University of New Mexico Health Sciences Center |
| Emily | Reese | University of New Mexico Health Sciences Center |
| Sarah | Ward | University of New Mexico Health Sciences Center |
| Kevin | Wilson | University of New Mexico Health Sciences Center |
| Amanda | Bogie | University of Oklahoma Health Sciences Center |
| Ryan | Brown | University of Oklahoma Health Sciences Center |
| Ryan | Butchee | University of Oklahoma Health Sciences Center |
| Gina | Fergeson | University of Oklahoma Health Sciences Center |
| Ryan | McKee | University of Oklahoma Health Sciences Center |
| Brandon | Mohler | University of Oklahoma Health Sciences Center |
| Tiffany | Moore | University of Oklahoma Health Sciences Center |
| Valorie | Owens | University of Oklahoma Health Sciences Center |
| Sarah | Stubbs | University of Oklahoma Health Sciences Center |
| Timothy | VanWagoner | University of Oklahoma Health Sciences Center |
| Kelly | Cowan | University of Vermont Medical Center |
| Meghan | Bethel | University of Vermont Medical Center |
| Laurie | Chassereau | University of Vermont Medical Center |
| Thomas | Lahiri | University of Vermont Medical Center |
| Lesley | Cottrell | West Virginia University |
| Lauren | Lake | West Virginia University |
| Kathy | Moffett | West Virginia University |
| Emily | Polak | West Virginia University |
| Sarah | Stutler | West Virginia University |
| Charlotte | Workman | West Virginia University |
| David | Warburton | Children’s Hospital of Los Angeles |
| Sindhu | Mohandas | Children’s Hospital of Los Angeles |
| John C. | Wood | Children’s Hospital of Los Angeles |
| Emma | Carpenter | Children’s Hospital of Los Angeles |
| Isabelle | Dhindsa | Children’s Hospital of Los Angeles |
| Samantha | Mejia | Children’s Hospital of Los Angeles |
| Nelly | Moghadam | Children’s Hospital of Los Angeles |
| Candice | Mulder | Children’s Hospital of Los Angeles |
| Sharon | O'Neil | Children’s Hospital of Los Angeles |
| Alisha | Osornio | Children’s Hospital of Los Angeles |
| Adrian | Rios | Children’s Hospital of Los Angeles |
| Sydney | Rosen | Children’s Hospital of Los Angeles |
| Andrea | Smith | Children’s Hospital of Los Angeles |
| Deeba | Tabibi | Children’s Hospital of Los Angeles |
| Sharon | Tang | Children’s Hospital of Los Angeles |
| Ariana | Teame | Children’s Hospital of Los Angeles |
| Melissa | Stockwell | Columbia University College of Physicians & Surgeons |
| Joshua | Milner | Columbia University College of Physicians & Surgeons |
| Erika B. | Rosenzweig | Columbia University College of Physicians & Surgeons |
| Brett | Anderson | Columbia University College of Physicians & Surgeons |
| Tawanda | Aquino | Columbia University College of Physicians & Surgeons |
| Elizabeth | Berg | Columbia University College of Physicians & Surgeons |
| Steve | Caddle | Columbia University College of Physicians & Surgeons |
| Marina | Catallozzi | Columbia University College of Physicians & Surgeons |
| Wendy | Chung | Columbia University College of Physicians & Surgeons |
| Tom | Connors | Columbia University College of Physicians & Surgeons |
| Aliva | De | Columbia University College of Physicians & Surgeons |
| Anny | Diaz Perez | Columbia University College of Physicians & Surgeons |
| Michael | DiLorenzo | Columbia University College of Physicians & Surgeons |
| Dani | Dumitriu | Columbia University College of Physicians & Surgeons |
| Kanwal | Farooqi | Columbia University College of Physicians & Surgeons |
| Michael | Fremed | Columbia University College of Physicians & Surgeons |
| Sylvie | Goldman | Columbia University College of Physicians & Surgeons |
| Kayla | Kaplan | Columbia University College of Physicians & Surgeons |
| Usha | Krishnan | Columbia University College of Physicians & Surgeons |
| Aimee | Layton | Columbia University College of Physicians & Surgeons |
| Angela | Lignelli-Dipple | Columbia University College of Physicians & Surgeons |
| Son | McClaren | Columbia University College of Physicians & Surgeons |
| Jonathan | Overdevest | Columbia University College of Physicians & Surgeons |
| Michelle | Rodriguez | Columbia University College of Physicians & Surgeons |
| Jay | Selman | Columbia University College of Physicians & Surgeons |
| Wendy | Silver | Columbia University College of Physicians & Surgeons |
| Raul | Silverio | Columbia University College of Physicians & Surgeons |
| Ana | Valdez de Romero | Columbia University College of Physicians & Surgeons |
| Celibell | Vargas | Columbia University College of Physicians & Surgeons |
| Alan | Werzberger | Best Healthcare Inc. |
| Daniella | Caputo | Best Healthcare Inc. |
| Camille | Leggieri | Best Healthcare Inc. |
| Pamela | Pretsch | Best Healthcare Inc. |
| Lawrence | Kleinman | Rutgers Robert Wood Johnson Medical School |
| Sunanda | Gaur | Rutgers Robert Wood Johnson Medical School |
| Lisa | Cerracchio | Rutgers Robert Wood Johnson Medical School |
| Amber | Folnagy | Rutgers Robert Wood Johnson Medical School |
| Maria | Gennaro | Rutgers Robert Wood Johnson Medical School |
| Sherri | Gzemski | Rutgers Robert Wood Johnson Medical School |
| Yue | Hao | Rutgers Robert Wood Johnson Medical School |
| Simon | Li | Rutgers Robert Wood Johnson Medical School |
| Sandee | Moroso | Rutgers Robert Wood Johnson Medical School |
| Manette | Ness-Cochinwala | Rutgers Robert Wood Johnson Medical School |
| Akhil | Patel | Rutgers Robert Wood Johnson Medical School |
| Benjamin | Richlin | Rutgers Robert Wood Johnson Medical School |
| Harsh | Sharma | Rutgers Robert Wood Johnson Medical School |
| Damaris | Soto | Rutgers Robert Wood Johnson Medical School |
| Christian | Suarez | Rutgers Robert Wood Johnson Medical School |
| Bibiana | Vargas Forero | Rutgers Robert Wood Johnson Medical School |
| Lynn | Olson | American Academy of Pediatrics |
| Kristin | Davis | American Academy of Pediatrics |
| Alexander | Fiks | American Academy of Pediatrics |
| Miranda | Griffith | American Academy of Pediatrics |
| Donna | Harris | American Academy of Pediatrics |
| Everly | Macario | American Academy of Pediatrics |
| Jennifer | Steffes | American Academy of Pediatrics |
| Alessandra | Torres | American Academy of Pediatrics |
| Rangaraj | Selvarangan | Children’s Mercy Kansas City |
| Dithi | Banerjee | Children’s Mercy Kansas City |
| Chris | Day | Children’s Mercy Kansas City |
| Kelsye | Howell | Children’s Mercy Kansas City |
| Megan | Mains | Children’s Mercy Kansas City |
| Juan C. | Salazar | Connecticut Children's Medical Center |
| William T. | Zempsky | Connecticut Children's Medical Center |
| Emily | Bean | Connecticut Children's Medical Center |
| Carlie | DeFelice | Connecticut Children's Medical Center |
| Hassan | El Chebib | Connecticut Children's Medical Center |
| Katherine | W. Herbst | Connecticut Children's Medical Center |
| Stephanie | Lesmes | Connecticut Children's Medical Center |
| Ian C. | Michelow | Connecticut Children's Medical Center |
| Melissa | Santos | Connecticut Children's Medical Center |
| Noah | Schulman | Connecticut Children's Medical Center |
| Wilson D. | Pace | DARTNet Institute |
| Alicia | Brooks-Greien | DARTNet Institute |
| Karinne | Colin | DARTNet Institute |
| Christina | M. Hester | DARTNet Institute |
| Ariadna | Juarez-Colunga | DARTNet Institute |
| Brian | Manning | DARTNet Institute |
| Joel | Shields | DARTNet Institute |
| Jack | Westfall | DARTNet Institute |
| Daphne | York | DARTNet Institute |
| Judy | Aschner | Hackensack Meridian Health Hospitals Corporation |
| Katharine | Clouser | Hackensack Meridian Health Hospitals Corporation |
| Justine | Griswold | Hackensack Meridian Health Hospitals Corporation |
| Donna | Lee | Hackensack Meridian Health Hospitals Corporation |
| Amanda | Nowakowski | Hackensack Meridian Health Hospitals Corporation |
| Maryellen | Riordan | Hackensack Meridian Health Hospitals Corporation |
| Hulya | Bukulmez | The MetroHealth System |
| David | Kaelber | The MetroHealth System |
| Rozina | Aamir | The MetroHealth System |
| Mohammed | Abuzahrieh | The MetroHealth System |
| Nandini | Bangalore | The MetroHealth System |
| Alexis | Brown | The MetroHealth System |
| Wendy | Dalton | The MetroHealth System |
| Suzanne | Fortuna | The MetroHealth System |
| Judi | Minium | The MetroHealth System |
| Bonnie | Rosolowski | The MetroHealth System |
| Sheila M. | Nolan | New York Medical College, Westchester Medical Center |
| Amal | Ahmed | New York Medical College, Westchester Medical Center |
| Suzanne | Braniecki | New York Medical College, Westchester Medical Center |
| Montserrat | Contreras | New York Medical College, Westchester Medical Center |
| Allen | Dozor | New York Medical College, Westchester Medical Center |
| Supriya | Jain | New York Medical College, Westchester Medical Center |
| Suzanne | Kaseta | New York Medical College, Westchester Medical Center |
| Sankaran | Krishnan | New York Medical College, Westchester Medical Center |
| Zachary | Messer | New York Medical College, Westchester Medical Center |
| Armando | Ramirez | New York Medical College, Westchester Medical Center |
| Aalok | Singh | New York Medical College, Westchester Medical Center |
| Randy | Williams | New York Medical College, Westchester Medical Center |
| Kyung | Rhee | Rady Children’s Hospital/University of California San Diego |
| Kelan | Tantisira | Rady Children’s Hospital/University of California San Diego |
| Almary | Akerlundh | Rady Children’s Hospital/University of California San Diego |
| Natacha | Akshoomoff | Rady Children’s Hospital/University of California San Diego |
| Maria | Arroyo | Rady Children’s Hospital/University of California San Diego |
| Wendy | Barrientos | Rady Children’s Hospital/University of California San Diego |
| Rakesh | Bhattacharjee | Rady Children’s Hospital/University of California San Diego |
| Bryant Y. | Chao | Rady Children’s Hospital/University of California San Diego |
| Maricela | Diaz | Rady Children’s Hospital/University of California San Diego |
| Sergio | Garcia | Rady Children’s Hospital/University of California San Diego |
| Sonia | Garcia | Rady Children’s Hospital/University of California San Diego |
| Guadalupe | Gomez | Rady Children’s Hospital/University of California San Diego |
| Trinidad | Herrera | Rady Children’s Hospital/University of California San Diego |
| Margarita | Holguin | Rady Children’s Hospital/University of California San Diego |
| Manaswitha | Khare | Rady Children’s Hospital/University of California San Diego |
| Elizabeth | Kiernan | Rady Children’s Hospital/University of California San Diego |
| Jeremy | Landeo Gutierrez | Rady Children’s Hospital/University of California San Diego |
| Ileana | Matta | Rady Children’s Hospital/University of California San Diego |
| Sofia | Reyes | Rady Children’s Hospital/University of California San Diego |
| Julie | Ryu | Rady Children’s Hospital/University of California San Diego |
| Cinthia | Sanchez | Rady Children’s Hospital/University of California San Diego |
| Andrea | Schreck | Rady Children’s Hospital/University of California San Diego |
| Megan R. | Warner | Rady Children’s Hospital/University of California San Diego |
| Uzma | Hasan | Saint Barnabas Medical Center, Newark Beth Israel |
| Elizabeth | Ricciardi | Saint Barnabas Medical Center, Newark Beth Israel |
| Vanessa | Trespalacios | Saint Barnabas Medical Center, Newark Beth Israel |
| Vince | Faustino | Yale School of Medicine |
| Matthew | Kluko | Yale School of Medicine |
| Carlos R. | Oliveira | Yale School of Medicine |
| Kyung E. | Rhee | University of California San Diego/Rady Children’s Hospital |
| Kelan G. | Tantisira | University of California San Diego/Rady Children’s Hospital |
| Almary | Akerlundh | University of California San Diego/Rady Children’s Hospital |
| Natacha | Akshoomoff | University of California San Diego/Rady Children’s Hospital |
| Wendy | Barrientos | University of California San Diego/Rady Children’s Hospital |
| Rakesh | Bhattacharjee | University of California San Diego/Rady Children’s Hospital |
| Bryant | Chao | University of California San Diego/Rady Children’s Hospital |
| Maricela | Diaz | University of California San Diego/Rady Children’s Hospital |
| Sonia | Garcia | University of California San Diego/Rady Children’s Hospital |
| Maria | Glenn-Arroyo | University of California San Diego/Rady Children’s Hospital |
| Guadalupe | Gomez | University of California San Diego/Rady Children’s Hospital |
| Trinidad | Herrera | University of California San Diego/Rady Children’s Hospital |
| Margarita | Holguin | University of California San Diego/Rady Children’s Hospital |
| Manaswitha | Khare | University of California San Diego/Rady Children’s Hospital |
| Elizabeth | A. Kiernan | University of California San Diego/Rady Children’s Hospital |
| Jeremy | Landeo-Gutierrez | University of California San Diego/Rady Children’s Hospital |
| Anika | Madan | University of California San Diego/Rady Children’s Hospital |
| Lisa | Ramos Vallejo | University of California San Diego/Rady Children’s Hospital |
| Sofia | Reyes | University of California San Diego/Rady Children’s Hospital |
| Julie | Ryu | University of California San Diego/Rady Children’s Hospital |
| Cinthia | E. Sanchez | University of California San Diego/Rady Children’s Hospital |
| Andrea | Schreck | University of California San Diego/Rady Children’s Hospital |
| Maira | Suarez | University of California San Diego/Rady Children’s Hospital |
| Megan R. | Warner | University of California San Diego/Rady Children’s Hospital |
| Patricia | Kinser | Virginia Commonwealth University |
| Amy | Salisbury | Virginia Commonwealth University |
| Jocelyn | Espinoza | Virginia Commonwealth University |
| Sara | Moyer | Virginia Commonwealth University |
| Amy | Rider | Virginia Commonwealth University |
| Sally | Russell | Virginia Commonwealth University |
| Michael | Schecter | Virginia Commonwealth University |
| Lindsey | Stevenson | Virginia Commonwealth University |
| Cheryl R. | Stein | New York University Grossman School of Medicine |
| Stephanie | V. Caldas | New York University Grossman School of Medicine |
| Thomas | Dylan Castro Ovalle | New York University Grossman School of Medicine |
| Anthony | Chung | New York University Grossman School of Medicine |
| Jonathan S | Farkas | New York University Grossman School of Medicine |
| Maria | Isidoro-Chino | New York University Grossman School of Medicine |
| Deniz | Kesebir | New York University Grossman School of Medicine |
| Eugenia | Kim | New York University Grossman School of Medicine |
| Ashley | Quarless | New York University Grossman School of Medicine |
| Alan | Schlechter | New York University Grossman School of Medicine |
| Ranjini | Srinivasan | New York University Grossman School of Medicine |
| Viren | D'Sa | Rhode Island Hospital |
| Fatoumata | Barry | Rhode Island Hospital |
| Phoebe | Burton | Rhode Island Hospital |
| Rosa | Cano Lorente | Rhode Island Hospital |
| Caroline | Cummins | Rhode Island Hospital |
| Stephanie | Wehbe | Rhode Island Hospital |
| Sandra | Brown | University of California San Diego |
| Anders | Dale | University of California San Diego |
| Terry | Jernigan | University of California San Diego |
| Kyung E. | Rhee | University of California San Diego |
| Susan | Tapert | University of California San Diego |
| Jose | Aguilar | University of California San Diego |
| David | Benjamin | University of California San Diego |
| Natalie C. | Buchbinder | University of California San Diego |
| Norma | Castro | University of California San Diego |
| Brandy | Emerson | University of California San Diego |
| Hugh | Garavan | University of California San Diego |
| Jennifer | Graves | University of California San Diego |
| Amanda | Guerrero | University of California San Diego |
| Janosch | Linkersdoerfer | University of California San Diego |
| Robert | Schooley | University of California San Diego |
| Wes | Thompson | University of California San Diego |
| Thanh | Trinh | University of California San Diego |
| Ron | Yang | University of California San Diego |
| Megan | Herting | Children's Hospital, Los Angeles |
| Elizabeth | Sowell | Children's Hospital, Los Angeles |
| Cynthia | Cisneros | Children's Hospital, Los Angeles |
| Lauren | Goedde | Children's Hospital, Los Angeles |
| Cedric | Manlhiot | Children's Hospital, Los Angeles |
| Raul | Gonzalez | Florida International University |
| Angela | Laird | Florida International University |
| Jorge | Limia | Florida International University |
| Robin | Aupperle | Laureate Institute for Brain Research |
| Martin | Paulus | Laureate Institute for Brain Research |
| Melanie | Curry | Laureate Institute for Brain Research |
| Nour S. | El-Sabbagh | Laureate Institute for Brain Research |
| Kevin | Gray | Medical University of South Carolina |
| Lindsay | Squeglia | Medical University of South Carolina |
| Samuel | Agbeh | Medical University of South Carolina |
| Cori | Herring | Medical University of South Carolina |
| Brittany | Mckenzie | Medical University of South Carolina |
| Bonnie | Nagel | Oregon Health & Science University |
| Abby | Espinoza | Oregon Health & Science University |
| Anthony | Hill | Oregon Health & Science University |
| Angie | Morales | Oregon Health & Science University |
| Fiona | Baker | SRI International |
| Eva | Muller-Oehring | SRI International |
| Ian | Colrain | SRI International |
| Ingrid | Durley | SRI International |
| Mirella | Dapretto | University of California, Los Angeles |
| Lucina | Uddin | University of California, Los Angeles |
| Susan | Bookheimer | University of California, Los Angeles |
| Christina | Caldera | University of California, Los Angeles |
| Jennifer | Dzul | University of California, Los Angeles |
| Cinthia | Zarate | University of California, Los Angeles |
| Marie | Banich | University of Colorado Boulder |
| Paola | Badilla | University of Colorado Boulder |
| Jennifer | Keith | University of Colorado Boulder |
| G | Kumar | University of Colorado Boulder |
| David | Messinger | University of Colorado Boulder |
| Katie | Prazak | University of Colorado Boulder |
| Sara Jo | Nixon | University of Florida |
| Melanie | Cardoso | University of Florida |
| Meagan | Sullivan | University of Florida |
| Stephen | Villard | University of Florida |
| Linda | Chang | University of Maryland Baltimore |
| Thomas | Ernst | University of Maryland Baltimore |
| Christine | Cloak | University of Maryland Baltimore |
| Huajun | Liang | University of Maryland Baltimore |
| Meghann C. | Ryan | University of Maryland Baltimore |
| Mary | Heitzerg | University of Michigan |
| Jennifer | Conley | University of Michigan |
| Leonard | Puttler | University of Michigan |
| Monica | Luciana | University of Minnesota |
| William | Iacono | University of Minnesota |
| Aaron | Schroeder | University of Minnesota |
| Duncan | Clark | University of Pittsburgh Medical Center |
| Doyeon (Dan) | Kim | University of Pittsburgh Medical Center |
| Megan | Retucci | University of Pittsburgh Medical Center |
| Edward | Freedman | University of Rochester |
| John | Foxe | University of Rochester |
| Sile | Ni Mhurchu | University of Rochester |
| Samantha | Spallina | University of Rochester |
| Erin | McGlade | University of Utah |
| Deborah | Yurgelun-Todd | University of Utah |
| Liz | Bell | University of Utah |
| Kirsten | Cline | University of Utah |
| Lauren | Heinrich | University of Utah |
| Perry | Renshaw | University of Utah |
| Hugh | Garavan | University of Vermont |
| Alexandra | Potter | University of Vermont |
| Cass | Barrett | University of Vermont |
| Sofia | Lozon | University of Vermont |
| Samantha | Spear | University of Vermont |
| Christine | Larson | University of Wisconsin |
| Krista | Lisdahl | University of Wisconsin |
| Tory | Clearwater | University of Wisconsin |
| Christine | Kaiver | University of Wisconsin |
| Caitlin | Nelson | University of Wisconsin |
| Zach | Paltzer | University of Wisconsin |
| Bridgette | Peteet | University of Wisconsin |
| James | Bjork | Virginia Commonwealth University |
| Mike | Neale | Virginia Commonwealth University |
| Olive | Calonge | Virginia Commonwealth University |
| Noah | Slattery | Virginia Commonwealth University |
| Lisa | Straub | Virginia Commonwealth University |
| Deanna | Barch | Washington University St. Louis |
| Andrew | Heath | Washington University St. Louis |
| Pamela | Madden | Washington University St. Louis |
| Taylor | Powell | Washington University St. Louis |
| Denise | Schmitz | Washington University St. Louis |
| Dylan | Gee | Yale University |
| Boris | Epie | Yale University |
| Rebekah | Hobbs | Yale University |
| Alex | Williams | Yale University |
| Zhouran (Rick) | Xiang | Yale University |
| Julie | Miller | Carelon Research |
| Jane | Newburger | Carelon Research |
| Felicia | Trachtenberg | Carelon Research |
| Ayesha | Amarnath | Carelon Research |
| James | Ambrosoli | Carelon Research |
| Denise | Artis | Carelon Research |
| Sachin | Bandari | Carelon Research |
| Emily | Birmingham | Carelon Research |
| Lozan | Eyob | Carelon Research |
| Kerri | Hayes | Carelon Research |
| Chenwei | Hu | Carelon Research |
| Melissa | Joyce | Carelon Research |
| Valentina | Kazlova | Carelon Research |
| Iris | Liu | Carelon Research |
| Amanda | Marshall | Carelon Research |
| Devine | Mbizdenyuy | Carelon Research |
| PJ | Mu | Carelon Research |
| Robin | Rowe | Carelon Research |
| Brooke | Sayles | Carelon Research |
| Mo | Zhang | Carelon Research |
| Pei-Ni | Jone | Ann & Robert Lurie Children's Hospital Chicago |
| Michael | Carr | Ann & Robert Lurie Children's Hospital Chicago |
| Lauren | Goodell | Ann & Robert Lurie Children's Hospital Chicago |
| Wantanabe | Kar | Ann & Robert Lurie Children's Hospital Chicago |
| Kathleen | Van't Hof | Ann & Robert Lurie Children's Hospital Chicago |
| Kristin | Sexson | Baylor College of Medicine/Texas Children's Hospital |
| Elias | Moussi | Baylor College of Medicine/Texas Children's Hospital |
| David | Olukayode | Baylor College of Medicine/Texas Children's Hospital |
| Sandra | Pena | Baylor College of Medicine/Texas Children's Hospital |
| Ricardo | Pignatelli | Baylor College of Medicine/Texas Children's Hospital |
| Faridis | Serrano | Baylor College of Medicine/Texas Children's Hospital |
| Sara | Sexson Tejtel | Baylor College of Medicine/Texas Children's Hospital |
| Lara | Shekerdemian | Baylor College of Medicine/Texas Children's Hospital |
| Audrey | Dionne | Boston Children's Hospital |
| Jane | Newburger | Boston Children's Hospital |
| Annette | Baker | Boston Children's Hospital |
| Sarah | DeFerrantini | Boston Children's Hospital |
| Thomas | Giorgio | Boston Children's Hospital |
| Numaira | Khan | Boston Children's Hospital |
| Simran | Mahanta | Boston Children's Hospital |
| MaryBeth | Son | Boston Children's Hospital |
| Matt | Oster | Children's Healthcare of Atlanta |
| Melissa | Burnett | Children's Healthcare of Atlanta |
| Kolby | Sanders-Lewis | Children's Healthcare of Atlanta |
| Suchitra | Rao | Children's Hospital of Colorado |
| Sonia | Chavez | Children's Hospital of Colorado |
| Georgia | Reis | Children's Hospital of Colorado |
| Jackie | Szmuszkovicz | Children's Hospital Los Angeles |
| Fariborz | Behzadian | Children's Hospital Los Angeles |
| Carla | Canas | Children's Hospital Los Angeles |
| Andrew | Cheng | Children's Hospital Los Angeles |
| Mike | Gawad | Children's Hospital Los Angeles |
| Paige | Johnson | Children's Hospital Los Angeles |
| Alicia | Kazarians | Children's Hospital Los Angeles |
| Sindhu | Mohandas | Children's Hospital Los Angeles |
| Anastasia | Sarkissian | Children's Hospital Los Angeles |
| Brandi | Scott | Children's Hospital Los Angeles |
| Consuelo | Secules | Children's Hospital Los Angeles |
| Jennifer | Su | Children's Hospital Los Angeles |
| Crystal | Vargas | Children's Hospital Los Angeles |
| Jodie | Votava-Smith | Children's Hospital Los Angeles |
| Sharon | Wagner-Lees | Children's Hospital Los Angeles |
| Shuo | Wang | Children's Hospital Los Angeles |
| Pierre | Wong | Children's Hospital Los Angeles |
| Yamuna | Sanil | Children's Hospital of Michigan |
| Sanjeev | Aggarwal | Children's Hospital of Michigan |
| Aiman | Almasnaah | Children's Hospital of Michigan |
| Nirupama | Kannikeswaran | Children's Hospital of Michigan |
| Kathleen | Meert | Children's Hospital of Michigan |
| Gautam | Singh | Children's Hospital of Michigan |
| Priya | Spencer | Children's Hospital of Michigan |
| Nancy | Sullivan | Children's Hospital of Michigan |
| Sureja | Sundaralingam | Children's Hospital of Michigan |
| Vishnu | Undyala | Children's Hospital of Michigan |
| Emily | Ward | Children's Hospital of Michigan |
| Amanda | Weber | Children's Hospital of Michigan |
| Charmaine | Williams Farr | Children's Hospital of Michigan |
| Tamara | Bradford | Children's Hospital of New Orleans |
| Marla | Johnston | Children's Hospital of New Orleans |
| Matthew | Elias | Children's Hospital of Philadelphia |
| Alex | Fiks | Children's Hospital of Philadelphia |
| Chris | Forrest | Children's Hospital of Philadelphia |
| Dana | Albizem | Children's Hospital of Philadelphia |
| Susan | Coffin | Children's Hospital of Philadelphia |
| Therese | Giglia | Children's Hospital of Philadelphia |
| Katherine | Lupton | Children's Hospital of Philadelphia |
| Grace | Marks | Children's Hospital of Philadelphia |
| Tonia | Morrison | Children's Hospital of Philadelphia |
| Shawn | O'Connor | Children's Hospital of Philadelphia |
| Daniel | Forsha | Children's Mercy Hospital |
| Jennifer | Nelson | Children's Mercy Hospital |
| Rachel | Sachdeva | Children's Mercy Hospital |
| Dara | Watkins | Children's Mercy Hospital |
| Ashraf S. | Harahsheh | Children's National Hospital |
| Jordyn | Britton | Children's National Hospital |
| Alix | Fetch | Children's National Hospital |
| Anita | Krishnan | Children's National Hospital |
| Onais | Tariq | Children's National Hospital |
| Sean | Lang | Cincinnati Children's Hospital Medical Center |
| Marisa | Almaguer | Cincinnati Children's Hospital Medical Center |
| Jim | Cnota | Cincinnati Children's Hospital Medical Center |
| Lauryn | Dugan | Cincinnati Children's Hospital Medical Center |
| Elise | Pickering | Cincinnati Children's Hospital Medical Center |
| Kathleen | Rathge | Cincinnati Children's Hospital Medical Center |
| Elizabeth | Mitchell | Cohen Children's Medical Center |
| Christine | Capone | Cohen Children's Medical Center |
| Nilanjana | Misra | Cohen Children's Medical Center |
| Olga | Shamailova | Cohen Children's Medical Center |
| Mark | Russell | CS Mott Children's Hospital/University of Michigan |
| Tammy | Doman | CS Mott Children's Hospital/University of Michigan |
| Lori | Harris | CS Mott Children's Hospital/University of Michigan |
| Keren | Hasbani | Dell Children's Medical Center |
| Sagar | Jani | Dell Children's Medical Center |
| Brian | McCrindle | Hospital for Sick Children/University of Toronto |
| Jessica | Bainton | Hospital for Sick Children/University of Toronto |
| Maryanne | Chrisant | Joe DiMaggio Children's Hospital |
| Doris | Alaby | Joe DiMaggio Children's Hospital |
| Norma | Barton | Joe DiMaggio Children's Hospital |
| Danielle | Katz | Joe DiMaggio Children's Hospital |
| Paulette | Smith | Joe DiMaggio Children's Hospital |
| Stephanie | Handler | Medical College of Wisconsin, Children's Hospital |
| Joe | Block | Medical College of Wisconsin, Children's Hospital |
| Regina | Cole | Medical College of Wisconsin, Children's Hospital |
| Jennifer | Maldonado | Medical College of Wisconsin, Children's Hospital |
| Kim | McHugh | Medical University of South Carolina |
| Andrew | Atz | Medical University of South Carolina |
| Megan | Bickford | Medical University of South Carolina |
| Jason | Buckley | Medical University of South Carolina |
| John | Costello | Medical University of South Carolina |
| Delany | Dennis | Medical University of South Carolina |
| Elizabeth | Emrath-Zwick | Medical University of South Carolina |
| Mary | Freeman | Medical University of South Carolina |
| Lanier | Jackson | Medical University of South Carolina |
| Madison | Johnson | Medical University of South Carolina |
| Tyler | Kasmarcak | Medical University of South Carolina |
| Elizabeth | Mack | Medical University of South Carolina |
| Scott | Pletzer | Medical University of South Carolina |
| Natasha | Ruth | Medical University of South Carolina |
| Carolyn | Taylor | Medical University of South Carolina |
| Sinai | Zyblewski | Medical University of South Carolina |
| Kanwal | Farooqi | Morgan Stanley Children's Hospital |
| Brett | Anderson | Morgan Stanley Children's Hospital |
| Katrina | Golub | Morgan Stanley Children's Hospital |
| Chanel | Rojas | Morgan Stanley Children's Hospital |
| Korsin | Rosalind | Morgan Stanley Children's Hospital |
| Chantal | Sanchez | Morgan Stanley Children's Hospital |
| Shubhika | Srivastava | Nemours, Alfred I. duPont Hospital for Children |
| Carol | Prospero | Nemours, Alfred I. duPont Hospital for Children |
| Deepika | Thacker | Nemours, Alfred I. duPont Hospital for Children |
| Ed | Williams | Nemours, Alfred I. duPont Hospital for Children |
| Varsha | Zadokar | Nemours, Alfred I. duPont Hospital for Children |
| Arash | Sabati | Phoenix Children's Hospital |
| Kylie | Domian | Phoenix Children's Hospital |
| Ashley | Herzberg | Phoenix Children's Hospital |
| Sanjana | Khanna | Phoenix Children's Hospital |
| Todd | Nowlen | Phoenix Children's Hospital |
| Susan | Park | Phoenix Children's Hospital |
| Jade | Porche | Phoenix Children's Hospital |
| Samantha | Stack | Phoenix Children's Hospital |
| Dongngan | Truong | Primary Children's Hospital/University of Utah |
| Andrea | Dunn | Primary Children's Hospital/University of Utah |
| Lilly | Fagatele | Primary Children's Hospital/University of Utah |
| Emma | Joyce | Primary Children's Hospital/University of Utah |
| Linda | Lambert | Primary Children's Hospital/University of Utah |
| Kirsten | Dummer | Rady Children's Hospital |
| Sherrie | Bandy | Rady Children's Hospital |
| Jane | Burns | Rady Children's Hospital |
| Katheryn | Crane | Rady Children's Hospital |
| Sanjeet | Hedge | Rady Children's Hospital |
| Joan | Pancheri | Rady Children's Hospital |
| Adriana | Tremoulet | Rady Children's Hospital |
| Ronald Mark | Payne | Riley Children's Hospital |
| Mary | Stumpf | Riley Children's Hospital |
| Michael | Portman | Seattle Children's Hospital |
| Hidemi | Kajimoto | Seattle Children's Hospital |
| Camden | Hebson | University of Alabama |
| Krissie | Hock | University of Alabama |
| Onyekachukwu | Osakwe | University of Mississippi |
| Jemylle | Morato | University of Mississippi |
| Divya | Shakti | University of Mississippi |
| Matt | Kadish | University of New Mexico |
| Hengameh | Raissy | University of New Mexico |
| Jerry | Larrabee | University of New Mexico |
| Kavita | Sharma | UT Southwestern, Children's Health Dallas |
| William | Anguiano | UT Southwestern, Children's Health Dallas |
| Catherine | Ikemba | UT Southwestern, Children's Health Dallas |
| Alejandra | Lozano | UT Southwestern, Children's Health Dallas |
| Maria | Martinez | UT Southwestern, Children's Health Dallas |
| Wendy | Rojas | UT Southwestern, Children's Health Dallas |
| Lerraughn | Morgan | Valley Children's Healthcare and Hospital |
| Isaura | Macias | Valley Children's Healthcare and Hospital |
| Carl | Owada | Valley Children's Healthcare and Hospital |
| Mayra | Rangel | Valley Children's Healthcare and Hospital |
| Michelle | Sykes | Valley Children's Healthcare and Hospital |
