# Supplementary material for: Ethnic and racial differences in children and young people with respiratory and neurological post-acute sequelae of SARS-CoV-2: an electronic health record-based cohort study from the RECOVER Initiative
Source: eClinicalMedicine. 2025 Jan 2;80:103042. doi: 10.1016/j.eclinm.2024.103042 (PMC11753962; doi:10.1016/j.eclinm.2024.103042)
Supplement: Supplemental Material Appendix 1 [file mmc3.docx]

**Supplemental Material Appendix 1**

**Exploring Ethnicity and Social Determinants of Health as Risk Factors for PASC: Findings from the RECOVER Initiative**

**Data Analysis Plan**

§01. Rationale

Little is known about the role of ethnicity and race and the impact of social determinants of health in the development of post-acute sequelae of SARS-CoV-2, aka “long COVID”, particularly in children. There have been several studies that have suggested that the prevalence of MIS-C and pediatric mortality may be higher in LatinX individuals. However, a broader interrogation of PASC is warranted. Our San Diego pediatric RECOVER site study, Pediatric Epidemiology and Disparities Study of Post-Acute SARS-CoV-2 (PEDS-PASS), and the RECOVER EHR cohort, have generated preliminary data that support an increased proportion of LatinX children (compared to the source population) developing both acute COVID-19 as well as COVID sequelae.

§02. Purpose and Aims

The **purpose of this study** is to compare the risk of post-acute sequelae of SARS CoV-2 in children by race, ethnicity, and social determinants of health. We hypothesize that the incidence of SARS-CoV-2 and respiratory PASC is greater in LatinX children, but that there may be other social determinants of health that may impact susceptibility, access to care and reporting.

The study has the following aims:

1. Determine the prevalence of general PASC, respiratory and neurologic sequelae following SARS-CoV-2 infection among different racial and ethnic groups.
2. Evaluate how factors such as social determinants of health and healthcare seeking behaviors may impact the prevalence in these populations.

§03. Milestones / Expected Output

§04. Study Sample / Cohort Definitions

Cases are defined as follows:

- patients who fit the computable phenotype (definite or probable) definition of PASC

Comparison groups will include the following:

- SARS-CoV-2 positive patients identified using viral and/or serology testing as well as SARS CoV-2 diagnosis codes.
- SARS-CoV-2 negative patients defined by presence of SARS CoV-2 negative viral test and no positive test or diagnosis for COVID, PASC, or MIS-C.
- Second comparison group without evidence of SARS-CoV-2 infection or long COVID code, with respiratory infection within the timeframe of interest

**Time Anchors**

The specific details of cohort formation are described below. Note that for dates, we use notation to indicate time, where day 0 refers to the day that a patient enters the cohort, a positive integer refers to the number of days after the cohort entrance that the observation period lasts, a negative integer refers to the number of days before the cohort entrance that the observation period lasts, oo refers to the end of the study period, and -oo refers to the beginning of the study period.

**Study period**: **Cohort entrance date** ([CED], [**0**]):

- Study period: March 1, 2020- August 1, 2022
- Cohort entrance date will be defined as date of earliest SARS-CoV-2 positive viral test or SARS-CoV-2 diagnosis. For patients diagnosed with PASC or MIS-C and no prior testing, if included, we can impute their cohort entrance date by picking a random day 28 - 90 days prior to impute their cohort entrance date as 28 days prior to earliest PASC diagnosis. For children with MIS-C, we can impute their cohort entrance date by picking a random day 14 - 42 days prior
- For SARS-CoV-2 negative patients, cohort entrance date may be defined as the date of the negative viral test (choose random date when a patient has more than one).
- **Follow-up window**: [28, 180 days] Diagnoses and other features used to identify PASC will be collected during the 28 day to 180 day post-acute period.
- **Inclusion criteria:**
  - Age < 21 years
  - Active patient population – at least 1 health care visit in the prior 12 months
  - Patient in PCORnet cohort
  - Cohort will include all types of healthcare visits, including ambulatory, inpatient, ED
  - Patients with at least 180 days of follow-up after index date.
- **Exclusion criteria**
  - Geocoding data unavailable
  - Site did not meet data quality standards
- **Attrition table:**

| *Attrition Criterion* | *N* | *Proportion of Total* |
| --- | --- | --- |
| *(A) All patients who meet eligibility criteria for inclusion in database* |  | *1.0* |
| *(B) Of A, patients who meet positive COVID-19 criteria* |  |  |
| *(C) Of A, patients who meet negative COVID-19 criteria* |  |  |
| *(D) Of B, patients who meet computable PASC definition* |  |  |
| *(E) Of C, patients who meet U09.9 PASC definition?* |  |  |
| *(F) Patients diagnosed with U09.9 (/B94.8) and non diagnosed with M35.81* |  |  |
| *(G) Patients diagnosed with MIS-C (M35.81)* |  |  |
| *(H) Of (D), patients with respiratory PASC* |  |  |
| 1. *Of (D), patients with neurologic PASC* |  |  |

§05. PCORnet RECOVER RECOVER Database

**PCORnet RECOVER Institutions**

All 40 PCORnet RECOVER sites

**PCORnet RECOVER Database Versions**

TBA

§06. Study Variables

Data elements (to use for summarization/outcomes/adjustment in analyses

- Age
- Sex
- Race
- Ethnicity
- Existence of existing chronic condition (could compute using Pediatric Medical Complexity Algorithm (PMCA)).
- Respiratory PMCA class
- Presence of and date of PASC, or PASC subphenotypes
- Variant periodate/month/time period of COVID infection/COVID variant.
- Acute COVID severity (Asymptomatic/Mild/Moderate/Severe—calculated from diagnoses, drug prescriptions, ICU and ventilator utilization during acute period)
- Hospitalization/ICU utilization at time of cohort entry
- Healthcare utilization prior to COVID-19 infection
- Social determinants of health indicators Area Deprivation Index (ADI) and Social Vulnerability index (SVI)
- Geography (national and regional origins), Rural/Urban

Outcomes of interest: definitions of PASC:

For PEDSnet/PCORnet analyses, main outcomes (bolded) are as follows:

1. SARS-CoV-2 infection (among patients with evidence of COVID - cases are SARS-CoV-2-positive, controls are test negative)
2. Computable phenotype definition of conclusive/probable PASC - See the following slides for definition of phenotype and diagnosis clusters that would be used to define subphenotypes below: [2023-07-phenotypes.pptx](https://nyulangone.sharepoint.com/:p:/r/sites/RECOVERPublicationsTeam2-EHRCollaborativeManuscripts/Shared%20Documents/EHR%20Collaborative%20Manuscripts/Ethnicity%20%26%20PASC/2023-07-phenotypes.pptx?d=wb05825c7420446629e8495054322b02b&csf=1&web=1&e=OV7ORd)
3. Respiratory manifestations of PASC (cases: patients with resp manifestations based on CP definition, comparison: patients who were test positive or diagnosed with COVID, second comparison: patients who were test negative)
4. Neurologic manifestations of PASC (cases: patients with neuro manifestations based on CP definition, comparison: patients who were test positive or diagnosed with COVID, second comparison: patients who were test negative)

Subanalyses, stratified analyses:

- Stratified analyses:
  - Stratify by age (< 5, 6-11, 12-17, 18-21)
  - Stratify by time period (ancestral, alpha, delta, omicron)
  - Stratify by SDOH outcomes of interest, (need to specify)
  - Stratify by comorbidities (PMCA severity, resp classification)
- Methodologically, will model this as interactions between race/ethnicity and each of the above covariates.
- Sensitivity analyses:
  - U09.9 diagnosis code
  - Computable phenotype definition of PASC excluding MIS-C
  - MIS-C only

§7. Analyses and Output

## 7.a. Descriptive Analyses

Summarize number and proportion of (a) patients with PASC, (b) SARS -CoV-2 positive, and (c) SARS-CoV-2 negative patients.

|  | Total | PASC positive | SARS-CoV-2 positive | SARS-CoV-2 negative | SMD |
| --- | --- | --- | --- | --- | --- |
| Age |  |  |  |  |  |
| Race |  |  |  |  |  |
| Ethnicity |  |  |  |  |  |
| Sex |  |  |  |  |  |
|  |  |  |  |  |  |
| PMCA |  |  |  |  |  |
| PMCA respiratory body system |  |  |  |  |  |
| Variant period |  |  |  |  |  |
| Location of testing (initial test) |  |  |  |  |  |
| Number of health care visits over certain time period (still need to define) |  |  |  |  |  |
| Geographical Region (Site?) |  |  |  |  |  |
| Urban/Rural (RUCA) |  |  |  |  |  |
| SDOH factors (Suggested: ADI/SVI) |  |  |  |  |  |

## 7.b. Statistical Modeling

Proposed analyses:

For outcomes of interest, compute OR of outcomes of interest among the following groups:

1. Ethnicity
2. Race
3. age
4. SDOH factors: ADI, SVI
5. Urban/Rural
6. High vs low healthcare utilization
7. PMCA classification
8. COVID severity
9. COVID variant (ancestral, alpha, delta, omicron)

Interaction between race/ethnicity and SDOH (ADI, SVI)
