# Supplementary material for: Ethnic and racial differences in children and young people with respiratory and neurological post-acute sequelae of SARS-CoV-2: an electronic health record-based cohort study from the RECOVER Initiative
Source: eClinicalMedicine. 2025 Jan 2;80:103042. doi: 10.1016/j.eclinm.2024.103042 (PMC11753962; doi:10.1016/j.eclinm.2024.103042)
Supplement: Supplemental Material PASC Ethnicity manuscript [file mmc4.docx]

**Supplemental Material Table of Contents**

|  | **Page** |
| --- | --- |
| **Supplemental Table 1.** Respiratory conditions codeset | 2 |
| **Supplemental Table 2.** Neurologic and Respiratory PASC codeset | 4 |
| **Supplemental Table 3.** Healthcare visit type and utilization cut-off points for each visit type | 14 |
| **Supplemental Table 4.** Definitions of Social Determinants of Health Indices | 15 |
| **Supplemental Table 5.** Acute COVID-19 Severity Classification | 16 |
| **Supplemental Figure 1.** CONSORT Diagram indicating eligible individuals for analyses | 17 |
| **Supplemental Table 6.** Sociodemographic characteristics SARS-CoV-2 positive and SARS CoV-2 negative respiratory infection group | 18 |
| **Supplemental Table 7.** Multivariable Logistic regression models evaluating covariates associated with respiratory manifestations known to be associated with PASC among a) SARS-CoV-2 positive cohort, b) SARS-CoV-2 negative cohort and c) SARS-CoV-2 acute respiratory infection cohort. | 21 |
| **Supplemental Table 8.** Multivariable Logistic regression models evaluating race and ethnicity groups associated with non-asthma respiratory manifestations known to be associated with PASC among the SARS-CoV-2 positive cohort | 23 |
| **Supplemental Table 9.** Multivariable logistic regression models evaluating covariates associated with respiratory PASC stratified by a) non severe acute SARS-CoV-2 infection and b) severe acute SARS-CoV-2 infection. | 24 |
| **Supplemental Table 10.** Respiratory PASC models stratified by area deprivation index | 27 |
| **Supplemental Table 11.** Respiratory PASC models stratified by underlying respiratory condition | 28 |
| **Supplemental Table 12.** Respiratory PASC models stratified by age | 29 |
| **Supplemental Table 13.** Respiratory PASC models including interaction between race/ethnicity and area deprivation index | 30 |
| **Supplemental Table 14.** Multivariable Logistic regression models evaluating covariates associated with neurological manifestations known to be associated with PASC among a) SARS-CoV-2 positive cohort, b) SARS-CoV-2 negative cohort and c) SARS-CoV-2 acute respiratory infection cohort. | 31 |
| **Supplemental Table 15.** Neurologic PASC models stratified by a) non severe acute SARS-CoV-2 infection and b) severe acute SARS-CoV-2 infection | 33 |
| **Supplemental Table 16.** Neurologic PASC models stratified by pre-existing neurologic condition | 36 |
| **Supplemental Figure 2.** Forest plot of Respiratory PASC models demonstrating OR and 95% CI for SVI subthemes | 37 |
| **Supplemental Figure 3.** Forest plot of Neurologic PASC models demonstrating OR and 95% CI for SVI subthemes | 38 |

**Supplemental Table 1.** Respiratory conditions codeset

| ***Concept Name*** | ***ICD-10-CM Code*** |
| --- | --- |
| Acute bronchiolitis | J21 |
| Acute bronchiolitis due to human metapneumovirus | J21.1 |
| Acute bronchiolitis due to other specified organisms | J21.8 |
| Acute bronchiolitis due to respiratory syncytial virus | J21.0 |
| Acute bronchiolitis, unspecified | J21.9 |
| Acute bronchitis due to Mycoplasma pneumoniae | J20.0 |
| Acute bronchitis due to parainfluenza virus | J20.4 |
| Acute bronchitis due to rhinovirus | J20.6 |
| Adenoviral pneumonia | J12.0 |
| Bacterial pneumonia, not elsewhere classified | J15 |
| Bacterial pneumonia, unspecified | J15.9 |
| Bronchopneumonia, unspecified | J18.0 |
| Human metapneumovirus pneumonia | J12.3 |
| Influenza due to identified seasonal influenza virus | J10 |
| Influenza due to identified zoonotic or pandemic influenza virus | J09 |
| Influenza with other manifestations, seasonal influenza virus identified | J10.8 |
| Influenza with other respiratory manifestations, seasonal influenza virus identified | J10.1 |
| Influenza with pneumonia, seasonal influenza virus identified | J10.0 |
| Lobar pneumonia, unspecified | J18.1 |
| Other bacterial pneumonia | J15.8 |
| Other pneumonia, organism unspecified | J18.8 |
| Other viral pneumonia | J12.8 |
| Parainfluenza virus pneumonia | J12.2 |
| Pneumonia due to Escherichia coli | J15.5 |
| Pneumonia due to Haemophilus influenzae | J14 |
| Pneumonia due to Klebsiella pneumoniae | J15.0 |
| Pneumonia due to Mycoplasma pneumoniae | J15.7 |
| Pneumonia due to other Gram-negative bacteria | J15.6 |
| Pneumonia due to other infectious organisms, not elsewhere classified | J16 |
| Pneumonia due to other specified infectious organisms | J16.8 |
| Pneumonia due to other streptococci | J15.4 |
| Pneumonia due to Streptococcus pneumoniae | J13 |
| Pneumonia due to streptococcus, group B | J15.3 |
| Pneumonia in bacterial diseases classified elsewhere | J17.0 |
| Pneumonia in diseases classified elsewhere | J17 |
| Pneumonia in mycoses | J17.2 |
| Pneumonia in other diseases classified elsewhere | J17.8 |
| Pneumonia in parasitic diseases | J17.3 |
| Pneumonia in viral diseases classified elsewhere | J17.1 |
| Pneumonia, organism unspecified | J18 |
| Pneumonia, unspecified | J18.9 |
| Respiratory syncytial virus pneumonia | J12.1 |
| Viral pneumonia, not elsewhere classified | J12 |
| Viral pneumonia, unspecified | J12.9 |
| Influenza with other manifestations, virus not identified | J11.8 |
| Influenza with other respiratory manifestations, virus not identified | J11.1 |
| Influenza with pneumonia, virus not identified | J11.0 |
| Influenza, virus not identified | J11 |
| Acute bronchitis | J20 |
| Acute bronchitis due to coxsackievirus | J20.3 |
| Acute bronchitis due to echovirus | J20.7 |
| Acute bronchitis due to other specified organisms | J20.8 |
| Acute bronchitis due to respiratory syncytial virus | J20.5 |
| Acute bronchitis due to streptococcus | J20.2 |
| Acute bronchitis, unspecified | J20.9 |
| Acute laryngitis | J04.0 |
| Acute laryngitis and tracheitis | J04 |
| Acute laryngopharyngitis | J06.0 |
| Acute laryngotracheitis | J04.2 |
| Acute nasopharyngitis [common cold] | J00 |
| Acute obstructive laryngitis [croup] | J05.0 |
| Acute pharyngitis | J02 |
| Acute pharyngitis due to other specified organisms | J02.8 |
| Acute pharyngitis, unspecified | J02.9 |
| Acute tracheitis | J04.1 |
| Acute upper respiratory infection, unspecified | J06.9 |
| Acute upper respiratory infections of multiple and unspecified sites | J06 |
| Bronchitis, not specified as acute or chronic | J40 |
| Mixed simple and mucopurulent chronic bronchitis | J41.8 |
| Other acute upper respiratory infections of multiple sites | J06.8 |

**Supplemental Table 2.** Neurologic and Respiratory PASC codeset

| ***Condition Name*** | ***ICD-10-CM Code*** | ***Codeset*** |
| --- | --- | --- |
| Expressive language disorder | F80.1 | Neurological |
| Other symptoms and signs involving cognitive functions and awareness | R41 | Neurological |
| Other amnesia | R41.3 | Neurological |
| Problems related to education and literacy, unspecified | Z55.9 | Neurological |
| Muscle weakness (generalized) | M62.81 | Neurological |
| Other malaise | R53.81 | Neurological |
| Other fatigue | R53.83 | Neurological |
| Postviral fatigue syndrome | G93.3 | Neurological |
| Other malaise and fatigue | R53.8 | Neurological |
| Chronic fatigue, unspecified | R53.82 | Neurological |
| Ophthalmoplegic migraine, intractable, with status migrainosus | G43.B11 | Neurological |
| Chronic tension-type headache | G44.22 | Neurological |
| Hemiplegic migraine | G43.4 | Neurological |
| Post-traumatic headache, unspecified | G44.30 | Neurological |
| Status migrainosus | G43.2 | Neurological |
| Drug-induced headache, not elsewhere classified, intractable | G44.41 | Neurological |
| Periodic headache syndromes in child or adult, intractable, with status migrainosus | G43.C11 | Neurological |
| Cyclical vomiting, not intractable, with status migrainosus | G43.A01 | Neurological |
| Chronic paroxysmal hemicrania, not intractable | G44.049 | Neurological |
| Other migraine, intractable | G43.81 | Neurological |
| Menstrual migraine, not intractable | G43.82 | Neurological |
| Other migraine, intractable | G43.891 | Neurological |
| Tension-type headache | G44.2 | Neurological |
| Headache, unspecified | R51.9 | Neurological |
| Hemiplegic migraine, intractable | G43.41 | Neurological |
| Migraine without aura, not intractable, without status migrainosus | G43.009 | Neurological |
| Persistent migraine aura without cerebral infarction, not intractable | G43.50 | Neurological |
| Migraine, unspecified | G43.9 | Neurological |
| Other migraine, not intractable | G43.899 | Neurological |
| Primary thunderclap headache | G44.53 | Neurological |
| Migraine with aura, intractable | G43.11 | Neurological |
| Drug-induced headache, not elsewhere classified, not intractable | G44.40 | Neurological |
| Other migraine | G43.8 | Neurological |
| Migraine with aura, not intractable | G43.10 | Neurological |
| Post-traumatic headache, unspecified, intractable | G44.301 | Neurological |
| Chronic cluster headache, intractable | G44.021 | Neurological |
| Persistent migraine aura with cerebral infarction, not intractable | G43.60 | Neurological |
| Episodic tension-type headache, not intractable | G44.219 | Neurological |
| Chronic cluster headache | G44.02 | Neurological |
| Chronic tension-type headache, not intractable | G44.229 | Neurological |
| Migraine, unspecified, not intractable | G43.90 | Neurological |
| Vascular headache, not elsewhere classified, not intractable | G44.10 | Neurological |
| Ophthalmoplegic migraine | G43.B | Neurological |
| Chronic migraine without aura, not intractable, without status migrainosus | G43.709 | Neurological |
| Drug-induced headache, not elsewhere classified | G44.4 | Neurological |
| Cyclical vomiting, intractable, with status migrainosus | G43.A11 | Neurological |
| Primary stabbing headache | G44.85 | Neurological |
| Complicated headache syndromes | G44.5 | Neurological |
| Post-traumatic headache | G44.3 | Neurological |
| Periodic headache syndromes in child or adult, not intractable | G43.C0 | Neurological |
| Headache | R51 | Neurological |
| Cluster headache syndrome, unspecified | G44.00 | Neurological |
| Cervicogenic headache | G44.86 | Neurological |
| Cyclical vomiting, in migraine, not intractable | G43.A0 | Neurological |
| Primary exertional headache | G44.84 | Neurological |
| Migraine, unspecified, not intractable, with status migrainosus | G43.901 | Neurological |
| Other migraine, not intractable, with status migrainosus | G43.801 | Neurological |
| Chronic migraine without aura, not intractable, with status migrainosus | G43.701 | Neurological |
| Chronic migraine without aura | G43.7 | Neurological |
| Menstrual migraine, not intractable, with status migrainosus | G43.D01 | Neurological |
| Ophthalmoplegic migraine, intractable, without status migrainosus | G43.B19 | Neurological |
| Short lasting unilateral neuralgiform headache with conjunctival injection and tearing (SUNCT) | G44.05 | Neurological |
| Cluster headaches and other trigeminal autonomic cephalgias (TAC) | G44.0 | Neurological |
| Chronic post-traumatic headache, intractable | G44.321 | Neurological |
| Other specified headache syndromes | G44.8 | Neurological |
| Persistent migraine aura with cerebral infarction, not intractable, without status migrainosus | G43.609 | Neurological |
| Hemiplegic migraine, not intractable, with status migrainosus | G43.401 | Neurological |
| Other migraine, intractable, with status migrainosus | G43.811 | Neurological |
| Periodic headache syndromes in child or adult, not intractable, with status migrainosus | G43.C01 | Neurological |
| Chronic paroxysmal hemicrania | G44.04 | Neurological |
| Chronic cluster headache, not intractable | G44.029 | Neurological |
| Hemiplegic migraine, intractable, without status migrainosus | G43.419 | Neurological |
| Other migraine, not intractable, without status migrainosus | G43.809 | Neurological |
| Migraine without aura, intractable, without status migrainosus | G43.019 | Neurological |
| Episodic cluster headache, not intractable | G44.019 | Neurological |
| Migraine without aura, intractable | G43.01 | Neurological |
| Menstrual migraine, intractable, with status migrainosus | G43.D11 | Neurological |
| Ophthalmoplegic migraine, not intractable, with status migrainosus | G43.B01 | Neurological |
| Ophthalmoplegic migraine, not intractable | G43.B0 | Neurological |
| Acute post-traumatic headache | G44.31 | Neurological |
| Menstrual migraine, intractable, without status migrainosus | G43.D19 | Neurological |
| Hemiplegic migraine, not intractable | G43.40 | Neurological |
| Chronic migraine without aura, intractable | G43.71 | Neurological |
| Short lasting unilateral neuralgiform headache with conjunctival injection and tearing (SUNCT), intractable | G44.051 | Neurological |
| Migraine with aura, not intractable, with status migrainosus | G43.101 | Neurological |
| Episodic tension-type headache, intractable | G44.211 | Neurological |
| Persistent migraine aura without cerebral infarction, intractable | G43.51 | Neurological |
| Tension-type headache, unspecified | G44.20 | Neurological |
| Migraine with aura, not intractable, without status migrainosus | G43.109 | Neurological |
| Hemicrania continua | G44.51 | Neurological |
| Periodic headache syndromes in child or adult, intractable | G43.C1 | Neurological |
| Chronic paroxysmal hemicrania, intractable | G44.041 | Neurological |
| Chronic post-traumatic headache | G44.32 | Neurological |
| Hemiplegic migraine, intractable, with status migrainosus | G43.411 | Neurological |
| Cyclical vomiting, in migraine, intractable | G43.A1 | Neurological |
| Acute post-traumatic headache, intractable | G44.311 | Neurological |
| Migraine, unspecified, not intractable, without status migrainosus | G43.909 | Neurological |
| Migraine with aura, intractable, with status migrainosus | G43.111 | Neurological |
| Episodic tension-type headache | G44.21 | Neurological |
| Migraine, unspecified, intractable, without status migrainosus | G43.919 | Neurological |
| Menstrual migraine, intractable, with status migrainosus | G43.831 | Neurological |
| Persistent migraine aura with cerebral infarction, intractable, without status migrainosus | G43.619 | Neurological |
| Primary cough headache | G44.83 | Neurological |
| Chronic migraine without aura, intractable, with status migrainosus | G43.711 | Neurological |
| Chronic post-traumatic headache, not intractable | G44.329 | Neurological |
| Episodic paroxysmal hemicrania, not intractable | G44.039 | Neurological |
| Cyclical vomiting, not intractable, without status migrainosus | G43.A09 | Neurological |
| Persistent migraine aura without cerebral infarction, intractable, without status migrainosus | G43.519 | Neurological |
| Persistent migraine aura with cerebral infarction, intractable | G43.61 | Neurological |
| Migraine without aura, intractable, with status migrainosus | G43.011 | Neurological |
| Periodic headache syndromes in child or adult, intractable, without status migrainosus | G43.C19 | Neurological |
| Post-traumatic headache, unspecified, not intractable | G44.309 | Neurological |
| Menstrual migraine, not intractable, with status migrainosus | G43.821 | Neurological |
| Other migraine, not intractable | G43.80 | Neurological |
| Episodic cluster headache | G44.01 | Neurological |
| Migraine without aura, not intractable | G43.00 | Neurological |
| Hypnic headache | G44.81 | Neurological |
| Migraine without aura | G43.0 | Neurological |
| Other complicated headache syndrome | G44.59 | Neurological |
| Tension-type headache, unspecified, not intractable | G44.209 | Neurological |
| Migraine with aura | G43.1 | Neurological |
| Persistent migraine aura with cerebral infarction, intractable, with status migrainosus | G43.611 | Neurological |
| Other trigeminal autonomic cephalgias (TAC), not intractable | G44.099 | Neurological |
| Headache with orthostatic component, not elsewhere classified | R51.0 | Neurological |
| Episodic cluster headache, intractable | G44.011 | Neurological |
| Chronic tension-type headache, intractable | G44.221 | Neurological |
| Other trigeminal autonomic cephalgias (TAC) | G44.09 | Neurological |
| Cluster headache syndrome, unspecified, intractable | G44.001 | Neurological |
| Menstrual migraine, not intractable, without status migrainosus | G43.D09 | Neurological |
| Other headache syndromes | G44 | Neurological |
| Migraine, unspecified, intractable, with status migrainosus | G43.911 | Neurological |
| Vascular headache, not elsewhere classified | G44.1 | Neurological |
| New daily persistent headache (NDPH) | G44.52 | Neurological |
| Hemiplegic migraine, not intractable, without status migrainosus | G43.409 | Neurological |
| Migraine with aura, intractable, without status migrainosus | G43.119 | Neurological |
| Persistent migraine aura without cerebral infarction, not intractable, without status migrainosus | G43.509 | Neurological |
| Migraine without aura, not intractable, with status migrainosus | G43.001 | Neurological |
| Episodic paroxysmal hemicrania | G44.03 | Neurological |
| Ophthalmoplegic migraine, intractable | G43.B1 | Neurological |
| Short lasting unilateral neuralgiform headache with conjunctival injection and tearing (SUNCT), not intractable | G44.059 | Neurological |
| Persistent migraine aura without cerebral infarction | G43.5 | Neurological |
| Menstrual migraine, not intractable, without status migrainosus | G43.829 | Neurological |
| Vascular headache, not elsewhere classified, intractable | G44.11 | Neurological |
| Periodic headache syndromes in child or adult, not intractable, without status migrainosus | G43.C09 | Neurological |
| Menstrual migraine, intractable | G43.83 | Neurological |
| Persistent migraine aura with cerebral infarction | G43.6 | Neurological |
| Migraine, unspecified, intractable | G43.91 | Neurological |
| Cluster headache syndrome, unspecified, not intractable | G44.009 | Neurological |
| Chronic migraine without aura, intractable, without status migrainosus | G43.719 | Neurological |
| Tension-type headache, unspecified, intractable | G44.201 | Neurological |
| Chronic migraine without aura, not intractable | G43.70 | Neurological |
| Persistent migraine aura with cerebral infarction, not intractable, with status migrainosus | G43.601 | Neurological |
| Persistent migraine aura without cerebral infarction, not intractable, with status migrainosus | G43.501 | Neurological |
| Other trigeminal autonomic cephalgias (TAC), intractable | G44.091 | Neurological |
| Migraine | G43 | Neurological |
| Acute post-traumatic headache, not intractable | G44.319 | Neurological |
| Persistent migraine aura without cerebral infarction, intractable, with status migrainosus | G43.511 | Neurological |
| Episodic paroxysmal hemicrania, intractable | G44.031 | Neurological |
| Menstrual migraine, intractable, without status migrainosus | G43.839 | Neurological |
| Other migraine, intractable, without status migrainosus | G43.819 | Neurological |
| Periodic headache syndromes in child or adult | G43.C | Neurological |
| Other headache syndrome | G44.89 | Neurological |
| Ophthalmoplegic migraine, not intractable, without status migrainosus | G43.B09 | Neurological |
| Tension-type headache | G44.2 | Neurological |
| Tension-type headache, unspecified | G44.20 | Neurological |
| Tension-type headache, unspecified, intractable | G44.201 | Neurological |
| Tension-type headache, unspecified, not intractable | G44.209 | Neurological |
| Episodic tension-type headache | G44.21 | Neurological |
| Episodic tension-type headache, intractable | G44.211 | Neurological |
| Episodic tension-type headache, not intractable | G44.219 | Neurological |
| Chronic tension-type headache | G44.22 | Neurological |
| Chronic tension-type headache, intractable | G44.221 | Neurological |
| Chronic tension-type headache, not intractable | G44.229 | Neurological |
| Insomnia | G47.0 | Neurological |
| Insomnia, unspecified | G47.00 | Neurological |
| Insomnia due to medical condition | G47.01 | Neurological |
| Other insomnia | G47.09 | Neurological |
| Narcolepsy and cataplexy | G47.4 | Neurological |
| Narcolepsy | G47.41 | Neurological |
| Narcolepsy with cataplexy | G47.411 | Neurological |
| Narcolepsy without cataplexy | G47.419 | Neurological |
| Narcolepsy in conditions classified elsewhere | G47.42 | Neurological |
| Narcolepsy in conditions classified elsewhere with cataplexy | G47.421 | Neurological |
| Narcolepsy in conditions classified elsewhere without cataplexy | G47.429 | Neurological |
| Parasomnia | G47.5 | Neurological |
| Parasomnia, unspecified | G47.50 | Neurological |
| Confusional arousals | G47.51 | Neurological |
| REM sleep behavior disorder | G47.52 | Neurological |
| Recurrent isolated sleep paralysis | G47.53 | Neurological |
| Parasomnia in conditions classified elsewhere | G47.54 | Neurological |
| Other parasomnia | G47.59 | Neurological |
| Sleep related movement disorders | G47.6 | Neurological |
| Periodic limb movement disorder | G47.61 | Neurological |
| Sleep related leg cramps | G47.62 | Neurological |
| Sleep related bruxism | G47.63 | Neurological |
| Other sleep related movement disorders | G47.69 | Neurological |
| Other sleep disorders | G47.8 | Neurological |
| Sleep disorder, unspecified | G47.9 | Neurological |
| Dizziness | R42 | Neurological |
| Acute respiratory distress syndrome | J80 | Respiratory |
| Cough | R05 | Respiratory |
| Acute cough | R05.1 | Respiratory |
| Cough syncope | R05.4 | Respiratory |
| Other specified cough | R05.8 | Respiratory |
| Cough, unspecified | R05.9 | Respiratory |
| Subacute cough | R05.2 | Respiratory |
| Chronic cough | R05.3 | Respiratory |
| Shortness of breath | R06.02 | Respiratory |
| Pleurisy | R09.1 | Respiratory |
| Hemoptysis | R04.2 | Respiratory |
| Periodic breathing | R06.3 | Respiratory |
| Hypoxemia | R09.02 | Respiratory |
| Hemorrhage from other sites in respiratory passages | R04.8 | Respiratory |
| Asphyxia and hypoxemia | R09.0 | Respiratory |
| Respiratory arrest | R09.2 | Respiratory |
| Stridor | R06.1 | Respiratory |
| Other specified symptoms and signs involving the circulatory and respiratory systems | R09.89 | Respiratory |
| Hemorrhage from other sites in respiratory passages | R04.89 | Respiratory |
| Hemorrhage from respiratory passages, unspecified | R04.9 | Respiratory |
| Mouth breathing | R06.5 | Respiratory |
| Sneezing | R06.7 | Respiratory |
| Other abnormalities of breathing | R06.8 | Respiratory |
| Dyspnea, unspecified | R06.00 | Respiratory |
| Hyperventilation | R06.4 | Respiratory |
| Wheezing | R06.2 | Respiratory |
| Abnormalities of breathing | R06 | Respiratory |
| Hemorrhage from throat | R04.1 | Respiratory |
| Apnea, not elsewhere classified | R06.81 | Respiratory |
| Hemorrhage from respiratory passages | R04 | Respiratory |
| Orthopnea | R06.01 | Respiratory |
| Abnormal sputum | R09.3 | Respiratory |
| Snoring | R06.83 | Respiratory |
| Epistaxis | R04.0 | Respiratory |
| Acute respiratory distress | R06.03 | Respiratory |
| Dyspnea | R06.0 | Respiratory |
| Other symptoms and signs involving the circulatory and respiratory system | R09 | Respiratory |
| Tachypnea, not elsewhere classified | R06.82 | Respiratory |
| Other specified symptoms and signs involving the circulatory and respiratory systems | R09.8 | Respiratory |
| Unspecified abnormalities of breathing | R06.9 | Respiratory |
| Hiccough | R06.6 | Respiratory |
| Nasal congestion | R09.81 | Respiratory |
| Other abnormalities of breathing | R06.89 | Respiratory |
| Other forms of dyspnea | R06.09 | Respiratory |
| Postnasal drip | R09.82 | Respiratory |
| Sleep apnea | G47.3 | Respiratory |
| Sleep apnea, unspecified | G47.30 | Respiratory |
| Obstructive sleep apnea (adult) (pediatric) | G47.33 | Respiratory |
| Idiopathic sleep related nonobstructive alveolar hypoventilation | G47.34 | Respiratory |
| Sleep related hypoventilation in conditions classified elsewhere | G47.36 | Respiratory |
| Central sleep apnea in conditions classified elsewhere | G47.37 | Respiratory |
| Other sleep apnea | G47.39 | Respiratory |

**Supplemental Table 3.** Healthcare visit type and utilization cut-off points for each visit type

| ***Visit Type*** | ***Cut-Off Points*** |
| --- | --- |
| **Outpatient Office** | None of visit type |
|  | < 3 visits |
|  | 3-5 visits |
|  | 6-9 visits |
|  | 9-15 visits |
|  | >15 visits |
| **Outpatient Test** | None of visit type |
|  | < 3 visits |
|  | 3-6 visits |
|  | 7-11 visits |
|  | 12-23 visits |
|  | >23 visits |
| **Other/Unknown** | None of visit type |
|  | 1-2 visits |
|  | 3-5 visits |
|  | 6-11 visits |
|  | >12 Visits |
| **Emergency Department** | None of visit type |
|  | 1-3 visits |
|  | >3 visits |
| **Emergency Department + Intensive Care Unit** | None of visit type |
|  | ED + ICU Visit |
| **Inpatient** | None of visit type |
|  | 1 visit |
|  | > 1 visits |
| **Inpatient + Intensive Care Unit** | None of visit type |
|  | Inpatient + ICU Visit |
| **Telehealth** | None of visit type |
|  | 1 visit |
|  | 2 – 4 visits |
|  | > 4 visits |

For outpatient visits, to interpret a patient’s outpatient healthcare utilization, the variable was categorized into quintiles based on the number of visits to an outpatient facility. Quintiles were chosen since it best captures the spread of the level of utilization. For inpatient visits, to interpret a patient’s inpatient healthcare utilization, the variable was categorized into 3 categories due to the rarity of inpatient utilization. 3 was chosen as more than 1 inpatient hospitalization is indicative of necessary care, 1 inpatient hospitalization is indicative of moderate need for care, and no inpatient utilization corresponds to a lower need for care.

**Supplemental Table 4.** Definitions of Social Drivers of Health Indices

| Index | Definition |
| --- | --- |
| **Area deprivation index (ADI):** | The Area Deprivation Index (ADI) is an index measuring neighborhood level socio-economic and environmental resources and exposures. Geocoded EHR data at the census block group level were linked to ADI in our analyses.  The ADI has been refined from a measure created by the Health Resources and Services Administration over 30 years ago. It includes the domains of income, education, employment, and housing quality and has been validated for neighborhood comparisons at the Census block group level. Predictive validity assessment documented correlations above 0.40 with health outcomes including all-cause mortality, infant mortality, and low birth weight. |
| **Social Vulnerability Index (SVI):** | Geocoded EHR data at the census tract group level were linked to data from an index measuring social vulnerability, known as the Social Vulnerability Index (SVI).  The SVI indicates the relative vulnerability of every U.S. Census tract and ranks the tracts on 16 different social factors including socioeconomic status, household characteristics, racial and ethnic minority status, housing type and transportation subthemes. SVI values range from 0 to 1 whereby the closer values are to 1, the higher the vulnerability. For more information on how vulnerability is defined and what factors are included in the calculation, please refer to the CDC/ATSDR SVI 2020 Documentation (<https://www.atsdr.cdc.gov/placeandhealth/svi/documentation/pdf/SVI2020Documentation_08.05.22.pdf>). |

**Supplemental Table 5.** Acute COVID-19 Severity Classification

| **Mild** | **Moderate** | **Severe** |
| --- | --- | --- |
| Abdominal pain | Bronchiolitis | Acute respiratory distress syndrome |
| Anorexia | Bronchitis | COVID-19–related severe sequelae |
| Cough | Gastroenteritis/dehydration | Death |
| COVID-19 diagnosis up to 13 d after test positivity | IV fluid administration in ED | ICU admission |
| Fatigue | Pneumonia | Mechanical ventilation |
| Fever and chills |  | Vasopressors and inotropes |
| Headache |  | Respiratory failure |
| Loss of taste or smell |  | Sepsis |
| Myalgia |  | Shock |
| Nasal congestion |  |  |
| Nausea, vomiting, or diarrhea |  |  |
| Respiratory symptoms |  |  |
| Sore throat |  |  |

From: Forrest et al. Pediatrics (2022) 149 (4): e2021055765

**Supplemental Figure 1.** CONSORT Diagram indicating eligible individuals for analyses


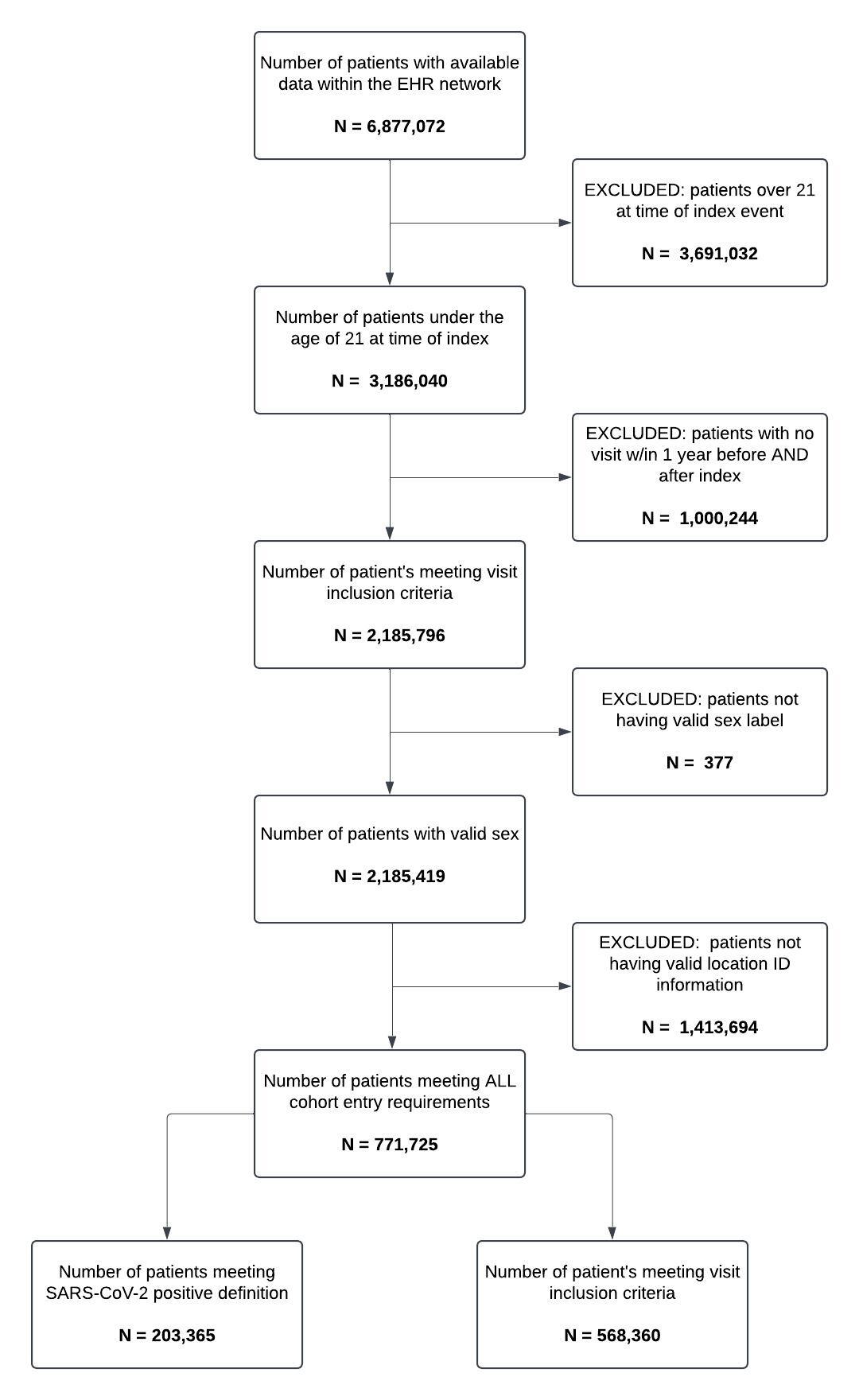


**Supplemental Table 6.** Sociodemographic characteristics SARS-CoV-2 positive and SARS CoV-2 negative respiratory infection group

| **Label** | **SARS-CoV-2 positive**  **N (%)** | **SARS-CoV-2 negative with respiratory infection**  **N (%)** | **SMD**  **(standard error)** |
| --- | --- | --- | --- |
|  | (N=203,365) | (N=152,901) |  |
| **Age Groups** |  |  | 0.378 (0.003) |
| **<5 years** | 67,291 (33.09 %) | 73,698 (48.20 %) |  |
| **12-17 years** | 59,494 (29.25 %) | 29,983 (19.61 %) |  |
| **6-11 years** | 53,343 (26.23 %) | 41,026 (26.83 %) |  |
| **18-21 years** | 23,237 (11.43 %) | 8,194 (5.36 %) |  |
| **Sex** |  |  | 0.014 (0.004) |
| **Female** | 101,289 (49.81 %) | 75,367 (49.29 %) |  |
| **Male** | 102,028 (50.17 %) | 77,517 (50.70 %) |  |
| **Other/unknown** | 48 (0.02 %) | 17 (0.01 %) |  |
| **Race ethnicity** |  |  | 0.056 (0.003) |
| **Hispanic Non-White** | 3,999 (1.97 %) | 2,865 (1.87 %) |  |
| **Hispanic White** | 25,866 (12.72 %) | 18,036 (11.80 %) |  |
| **Non-Hispanic Asian** | 7,985 (3.93 %) | 5,699 (3.73 %) |  |
| **Non-Hispanic Black** | 30,853 (15.17 %) | 22,793 (14.91 %) |  |
| **Non-Hispanic Multiple Races** | 4,085 (2.01 %) | 4,037 (2.64 %) |  |
| **Non-Hispanic Other** | 290 (0.14 %) | 213 (0.14 %) |  |
| **Non-Hispanic White** | 84,201 (41.40 %) | 62,948 (41.17 %) |  |
| **Other/Unknown** | 46,086 (22.66 %) | 36,310 (23.75 %) |  |
| **Index Event Period** |  |  | 0.624 (0.003) |
| **Mar-May 2020** | 2,123 (1.04 %) | 1,843 (1.21 %) |  |
| **Jun-Aug 2020** | 6,608 (3.25 %) | 4,944 (3.23 %) |  |
| **Sep-Nov 2020** | 11,207 (5.51 %) | 10,605 (6.94 %) |  |
| **Dec-Feb 2021** | 23,689 (11.65 %) | 9,764 (6.39 %) |  |
| **Mar-May 2021** | 12,235 (6.02 %) | 13,293 (8.69 %) |  |
| **Jun-Aug 2021** | 12,915 (6.35 %) | 16,347 (10.69 %) |  |
| **Sep-Nov 2021** | 23,033 (11.33 %) | 33,746 (22.07 %) |  |
| **Dec-Feb 2022** | 75,366 (37.06 %) | 24,863 (16.26 %) |  |
| **Mar-May 2022** | 17,384 (8.55 %) | 25,746 (16.84 %) |  |
| **Jun-Jul 2022** | 18,805 (9.25 %) | 11,750 (7.68 %) |  |
| **SARS-CoV-2 Infection Severity** |  |  | 3.302 (0.005) |
| **Asymptomatic** | 31,518 (15.50 %) | 152,901 (100.00 %) |  |
| **Mild** | 101,575 (49.95 %) | 0 (0.00 %) |  |
| **Moderate** | 53,647 (26.38 %) | 0 (0.00 %) |  |
| **Severe** | 16,625 (8.17 %) | 0 (0.00 %) |  |
| **Location of index visit** |  |  | 0.516 (0.003) |
| **ED** | 28,258 (13.90 %) | 35,841 (23.44 %) |  |
| **Inpatient** | 4,406 (2.17 %) | 5,332 (3.49 %) |  |
| **Other/Unknown** | 27,210 (13.38 %) | 14,475 (9.47 %) |  |
| **Outpatient Office** | 75,931 (37.34 %) | 73,202 (47.88 %) |  |
| **Outpatient: Test Only** | 42,760 (21.03 %) | 19,715 (12.89 %) |  |
| **Telehealth** | 24,800 (12.19 %) | 4,336 (2.84 %) |  |
| **Degree of Chronicity (PMCA)** |  |  | 0.081 (0.003) |
| **Non-Chronic** | 180,432 (88.72 %) | 139,257 (91.08 %) |  |
| **Chronic** | 10,776 (5.30 %) | 6,867 (4.49 %) |  |
| **Complex Chronic** | 12,157 (5.98 %) | 6,777 (4.43 %) |  |
| **Patients with chronic respiratory/pulmonary conditions** |  |  | 0.047 (0.003) |
| **Non-Chronic** | 193,573 (95.19 %) | 146,904 (96.08 %) |  |
| **Chronic** | 5,265 (2.59 %) | 3,490 (2.28 %) |  |
| **Complex Chronic** | 4,527 (2.23 %) | 2,507 (1.64 %) |  |
| **Patients with chronic neurological conditions** |  |  | 0.051 (0.003) |
| **Non-Chronic** | 196,150 (96.45 %) | 148,724 (97.27 %) |  |
| **Chronic** | 1,719 (0.85 %) | 1,209 (0.79 %) |  |
| **Complex Chronic** | 5,496 (2.70 %) | 2,968 (1.94 %) |  |
| **ADI National Rank** |  |  | 0.03 (0.003) |
| **0-19th percentile** | 34,387 (16.91 %) | 24,649 (16.12 %) |  |
| **20-39th percentile** | 57,849 (28.45 %) | 45,240 (29.59 %) |  |
| **40-59th percentile** | 43,325 (21.30 %) | 32,684 (21.38 %) |  |
| **60-79th percentile** | 35,743 (17.58 %) | 26,590 (17.39 %) |  |
| **80-100th percentile** | 32,061 (15.77 %) | 23,738 (15.53 %) |  |
| **Social Vulnerability Index** |  |  | 0.008 (0.003) |
| **0-25th percentile** | 57,063 (28.06 %) | 42,705 (27.93 %) |  |
| **25-50th percentile** | 43,413 (21.35 %) | 32,420 (21.20 %) |  |
| **50-75th percentile** | 41793 (20.55 %) | 31,874 (20.85 %) |  |
| **75-100th percentile** | 60,968 (29.98 %) | 45,801 (29.95 %) |  |
| **Missing or Suppressed ADI value** | 128 (0.06 %) | 101 (0.07 %) |  |
| **PASC** | 10,969 (5.39 %) | 0 (0.00 %) | 0.338 (0.003) |
| **No PASC** | 192,396 (94.61 %) | 152,901 (100.00 %) |  |
| **Patients with Neurological presentation** | 2,009 (0.99 %) | 1,112 (0.73 %) | 0.031 (0.003) |
| **Patients with Respiratory presentation** | 3,217 (1.58 %) | 2,116 (1.38 %) | 0.015 (0.003) |

^a^SMD- standardized mean difference

^b^ADI- Area Deprivation Index (ADI) is a measure of social vulnerability that ranks census block groups by socioeconomic disadvantage in an area of interest and is calculated using factors such as income, education, employment, and housing quality. A block group with a ranking of 1 indicates the lowest level of “disadvantage” within the nation and an ADI with a ranking of 100 indicates the highest level of “disadvantage”.

Abbreviations:

PMCA- pediatric medical complexity algorithm

PASC- post acute sequelae of SARS-CoV-2

ED-emergency department

SVI- Social vulnerability index

**Supplemental Table 7.** Multivariable Logistic regression models evaluating covariates associated with respiratory manifestations known to be associated with PASC among a) SARS-CoV-2 positive cohort, b) SARS-CoV-2 negative cohort and c) SARS-CoV-2 acute respiratory infection cohort.

| **Variable Label** | **Odds Ratio Positive Cohort (2.5%, 97.5%)** | **Odds Ratio Negative Cohort (2.5%, 97.5%)** | **Odds Ratio Respiratory Cohort (2.5%, 97.5%)** |
| --- | --- | --- | --- |
| **Age Group (REF = 6-11)** | | | |
| **<5** | 1.783 (1.619,1.964) | 1.806 (1.69,1.931) | 1.814 (1.605,2.05) |
| **12-17** | 0.702 (0.625,0.788) | 0.807 (0.744,0.875) | 0.74 (0.624,0.876) |
| **18-20** | 0.696 (0.589,0.822) | 0.659 (0.578,0.752) | 0.728 (0.547,0.97) |
| **Race/Ethnicity (REF = Non-Hispanic White)** | | | |
| **Hispanic Non-White** | 1.009 (0.783,1.299) | 1.176 (0.995,1.391) | 1.527 (1.176,1.981) |
| **Hispanic White** | 1.21 (1.067,1.372) | 1.062 (0.964,1.169) | 0.917 (0.776,1.083) |
| **Non-Hispanic Asian** | 1.205 (0.996,1.456) | 0.942 (0.827,1.072) | 1.051 (0.843,1.311) |
| **Non-Hispanic Black** | 0.998 (0.893,1.114) | 0.964 (0.89,1.045) | 0.923 (0.804,1.059) |
| **Non-Hispanic Multiple Races** | 0.983 (0.771,1.254) | 1.215 (1.056,1.398) | 1.121 (0.871,1.443) |
| **Other/Unknown** | 1.044 (0.944,1.154) | 1.009 (0.942,1.081) | 0.976 (0.867,1.1) |
| **ADI (REF = 0-19th Percentile)** | | | |
| **20-39th percentile** | 1.033 (0.93,1.146) | 1.007 (0.939,1.08) | 1.014 (0.896,1.149) |
| **40-59th percentile** | 1.087 (0.97,1.217) | 1.145 (1.06,1.237) | 1.217 (1.065,1.391) |
| **60-79th percentile** | 1.15 (1.021,1.295) | 1.165 (1.071,1.267) | 1.167 (1.009,1.35) |
| **80-100th percentile** | 0.994 (0.87,1.135) | 1.127 (1.026,1.237) | 1.052 (0.895,1.237) |
| **Period of Index Event (REF = Mar-May 2020)** | | | |
| **Jun-Aug 2020** | 2.017 (1.272,3.199) | 1.01 (0.843,1.211) | 1.168 (0.759,1.799) |
| **Sep-Nov 2020** | 1.916 (1.235,2.974) | 1.072 (0.898,1.279) | 1.377 (0.926,2.046) |
| **Dec-Feb 2021** | 2.072 (1.363,3.151) | 1.305 (1.096,1.555) | 1.427 (0.96,2.122) |
| **Mar-May 2021** | 2.159 (1.402,3.325) | 1.318 (1.108,1.567) | 1.594 (1.085,2.342) |
| **Jun-Aug 2021** | 3.916 (2.57,5.965) | 1.487 (1.252,1.767) | 1.86 (1.275,2.713) |
| **Sep-Nov 2021** | 3.592 (2.374,5.436) | 1.641 (1.388,1.94) | 1.827 (1.261,2.646) |
| **Dec-Feb 2022** | 3.214 (2.14,4.826) | 1.75 (1.478,2.071) | 1.94 (1.336,2.818) |
| **Mar-Jul 2022** | 0.593 (0.376,0.936) | 0.688 (0.57,0.83) | 0.665 (0.449,0.985) |
| **PMCA - Respiratory (REF = Non-Chronic)** | | | |
| **Chronic** | 2.093 (1.786,2.452) | 2.148 (1.911,2.415) | 1.474 (1.17,1.855) |
| **Complex Chronic** | 3.278 (2.903,3.7) | 3.883 (3.6,4.189) | 2.581 (2.177,3.062) |

^a^SMD- standardized mean difference

^b^ADI- Area Deprivation Index (ADI) is a measure of social vulnerability that ranks census block groups by socioeconomic disadvantage in an area of interest and is calculated using factors such as income, education, employment, and housing quality. A block group with a ranking of 1 indicates the lowest level of “disadvantage” within the nation and an ADI with a ranking of 100 indicates the highest level of “disadvantage”.

^C^ SVI- Social vulnerability index

**Supplemental Table 8.** Multivariable Logistic regression models evaluating race and ethnicity groups associated with non-asthma respiratory manifestations known to be associated with PASC among the SARS-CoV-2 positive cohort

| **Race/Ethnicity** | **Odds Ratio (95% CI)** |
| --- | --- |
| Other/Unknown | 0.979 (0.730, 1.312) |
| NH-Multiple Race | 0.980 (0.750, 1.282) |
| NH-Black | 1.010 (0.891, 1.145) |
| NH-Asian | 1.236 (1.012, 1.510) |
| Hispanic White | 1.1239 (1.012, 1.422) |
| Hispanic Non-White | 0.979 (0.730, 1.312) |

**Supplemental Table 9**. Multivariable Logistic regression models evaluating covariates associated with respiratory PASC stratified by a) non severe acute SARS-CoV-2 infection and b) severe acute SARS-CoV-2 infection.

1. Non-severe

| **Variable Label** | **Odds Ratio (2.5%, 97.5%)** |
| --- | --- |
| **Age Group (REF = 6-11)** | |
| **<5** | **1.397 (1.19, 1.641)** |
| **12-17** | **0.702 (0.587, 0.84)** |
| **18-20** | **0.639 (0.497, 0.822)** |
| **Race/Ethnicity (REF = Non-Hispanic White)** | |
| **Hispanic Non-White** | 0.747 (0.462, 1.208) |
| **Hispanic White** | 1.155 (0.945, 1.411) |
| **Non-Hispanic Asian** | 1.209 (0.883, 1.656) |
| **Non-Hispanic Black** | 1.106 (0.918, 1.333) |
| **Non-Hispanic Multiple Races** | 1.119 (0.734, 1.705) |
| **Other/Unknown** | 1.122 (0.951, 1.325) |
| **ADI (REF = 0-19th Percentile)** | |
| **20-39th percentile** | **1.245 (1.047, 1.48)** |
| **40-59th percentile** | **1.22 (1.004, 1.482)** |
| **60-79th percentile** | **1.339 (1.095, 1.636)** |
| **80-100th percentile** | 1.226 (0.984, 1.528) |
| **Period of Index Event (REF = Mar-May 2020)** | |
| **Jun-Aug 2020** | **4.098 (1.252, 13.408)** |
| **Sep-Nov 2020** | **3.25 (1, 10.56)** |
| **Dec-Feb 2021** | **3.794 (1.197, 12.025)** |
| **Mar-May 2021** | **4.229 (1.314, 13.603)** |
| **Jun-Aug 2021** | **6.978 (2.195, 22.189)** |
| **Sep-Nov 2021** | **6.958 (2.204, 21.968)** |
| **Dec-Feb 2022** | **6.773 (2.163, 21.204)** |
| **Mar-Jul 2022** | 0.919 (0.27, 3.128) |
| **PMCA - Respiratory (REF = Non-Chronic)** | |
| **Chronic** | **2.305 (1.682, 3.159)** |
| **Complex Chronic** | **3.574 (2.631, 4.854)** |

1. Severe

| **Variable Label** | **Odds Ratio (2.5%, 97.5%)** |
| --- | --- |
| **Age Group (REF = 6-11)** | |
| **<5** | **2.043 (1.809, 2.306)** |
| **12-17** | **0.74 (0.635, 0.863)** |
| **18-20** | 0.82 (0.656, 1.026) |
| **Race/Ethnicity (REF = Non-Hispanic White)** | |
| **Hispanic Non-White** | 1.263 (0.934, 1.708) |
| **Hispanic White** | **1.365 (1.159, 1.609)** |
| **Non-Hispanic Asian** | **1.279 (1.006, 1.626)** |
| **Non-Hispanic Black** | 1.005 (0.875, 1.154) |
| **Non-Hispanic Multiple Races** | 0.943 (0.701, 1.269) |
| **Other/Unknown** | 1.081 (0.953, 1.227) |
| **ADI (REF = 0-19th Percentile)** | |
| **20-39th percentile** | 0.907 (0.795, 1.035) |
| **40-59th percentile** | 0.991 (0.861, 1.14) |
| **60-79th percentile** | 1.033 (0.89, 1.199) |
| **80-100th percentile** | 0.872 (0.738, 1.031) |
| **Period of Index Event (REF = Mar-May 2020)** | |
| **Jun-Aug 2020** | **1.79 (1.052, 3.046)** |
| **Sep-Nov 2020** | **1.878 (1.154, 3.054)** |
| **Dec-Feb 2021** | **1.949 (1.231, 3.087)** |
| **Mar-May 2021** | **1.899 (1.179, 3.059)** |
| **Jun-Aug 2021** | **3.729 (2.349, 5.918)** |
| **Sep-Nov 2021** | **3.19 (2.029, 5.015)** |
| **Dec-Feb 2022** | **2.735 (1.759, 4.254)** |
| **Mar-Jul 2022** | **0.562 (0.342, 0.926)** |
| **PMCA - Respiratory (REF = Non-Chronic)** | |
| **Chronic** | **1.802 (1.5, 2.166)** |
| **Complex Chronic** | **2.917 (2.551, 3.334)** |

^a^ADI- Area Deprivation Index (ADI) is a measure of social vulnerability that ranks census block groups by socioeconomic disadvantage in an area of interest and is calculated using factors such as income, education, employment, and housing quality. A block group with a ranking of 1 indicates the lowest level of “disadvantage” within the nation and an ADI with a ranking of 100 indicates the highest level of “disadvantage”.

**Supplemental Table 10.** Respiratory PASC Models stratified by area deprivation index

| **Variable Label** | **0-19th Percentile Odds Ratio** | **20-39th Percentile Odds Ratio** | **40-59th Percentile Odds Ratio** | **60-79th Percentile Odds Ratio** | **80-100th Percentile Odds Ratio** |
| --- | --- | --- | --- | --- | --- |
| **Race/Ethnicity - Hispanic Non-White** | 0.808 (0.432,1.51) | 0.932 (0.567,1.53) | 0.886 (0.501,1.569) | 1.009 (0.532,1.912) | 1.657 (0.913,3.007) |
| **Race/Ethnicity - Hispanic White** | 1.078 (0.828,1.403) | **1.322 (1.049,1.667)** | 1.288 (0.975,1.702) | 1.021 (0.729,1.43) | 1.357 (0.918,2.008) |
| **Race/Ethnicity - Non-Hispanic Asian** | 1.204 (0.897,1.615) | 1.262 (0.867,1.835) | 1.153 (0.698,1.904) | 1.182 (0.635,2.199) | 0.547 (0.169,1.766) |
| **Race/Ethnicity - Non-Hispanic Black** | 0.968 (0.714,1.312) | 0.974 (0.762,1.245) | 1.016 (0.79,1.306) | 1.167 (0.924,1.473) | 1.002 (0.77,1.304) |
| **Race/Ethnicity - Non-Hispanic Multiple Races** | 0.753 (0.389,1.459) | 0.938 (0.577,1.523) | 1.001 (0.625,1.604) | 1.186 (0.698,2.015) | 0.917 (0.448,1.878) |
| **Race/Ethnicity - Other/Unknown** | 1.1 (0.907,1.333) | 1.014 (0.834,1.234) | 0.94 (0.74,1.195) | 1.065 (0.81,1.4) | 1.114 (0.812,1.529) |

**Supplemental Table 11.** Respiratory PASC models stratified by presence of an underlying respiratory condition

| **Variable Label** | **Non-Chronic Odds Ratio** | **Chronic Odds Ratio** | **Complex Chronic Odds Ratio** |
| --- | --- | --- | --- |
| **Race/Ethnicity - Hispanic Non-White** | 0.995 (0.736,1.345) | 1.396 (0.621,3.136) | 0.929 (0.529,1.63) |
| **Race/Ethnicity - Hispanic White** | **1.19 (1.028,1.378)** | **1.6 (1.039,2.465)** | 1.253 (0.93,1.688) |
| **Race/Ethnicity - Non-Hispanic Asian** | 1.179 (0.946,1.471) | 1.495 (0.72,3.101) | 1.153 (0.758,1.754) |
| **Race/Ethnicity - Non-Hispanic Black** | 1.041 (0.913,1.188) | 0.913 (0.625,1.334) | 1.052 (0.831,1.333) |
| **Race/Ethnicity - Non-Hispanic Multiple Races** | 0.944 (0.699,1.275) | 1.263 (0.568,2.808) | 1.049 (0.652,1.687) |
| **Race/Ethnicity - Other/Unknown** | 1.019 (0.906,1.147) | 1.258 (0.866,1.827) | 1.106 (0.891,1.374) |

**Supplemental Table 12.** Models of Respiratory PASC stratified by age

| **Variable Label** | **<5 years Odds Ratio** | **6-11 years Odds Ratio** | **12-17 years Odds Ratio** | **18-21 years Odds Ratio** |
| --- | --- | --- | --- | --- |
| **Race/Ethnicity - Hispanic Non-White** | 0.929 (0.663,1.301) | 1.136  (0.645,2) | 1.127 (0.627,2.027) | 0.709 (0.215,2.34) |
| **Race/Ethnicity - Hispanic White** | **1.235 (1.037,1.471)** | **1.505 (1.169,1.937)** | 0.921 (0.674,1.259) | 0.777 (0.464,1.301) |
| **Race/Ethnicity - Non-Hispanic Asian** | 1.111 (0.871,1.418) | 1.408 (0.936,2.12) | 1.061 (0.615,1.83) | 1.638 (0.756,3.547) |
| **Race/Ethnicity - Non-Hispanic Black** | 1.025 (0.884,1.19) | 0.982 (0.768,1.256) | 0.989 (0.766,1.278) | 0.74 (0.477,1.149) |
| **Race/Ethnicity - Non-Hispanic Multiple Races** | 0.846 (0.608,1.176) | 1.113 (0.681,1.818) | 1.252 (0.696,2.255) | 1.362 (0.41,4.52) |
| **Race/Ethnicity - Other/Unknown** | 0.921 (0.805,1.055) | **1.404 (1.135,1.736)** | 1.032 (0.812,1.312) | 0.981 (0.653,1.473) |

**Supplemental Table 13**. Respiratory PASC models including interaction between race/ethnicity and area deprivation index

|  | **Odds ratio (95% CI)** |
| --- | --- |
| **Hispanic Non-White :   20-39th percentile** | 1.194 (0.539,2.643) |
| **Hispanic White :   20-39th percentile** | 1.097 (0.785,1.533) |
| **Non-Hispanic Asian :   20-39th percentile** | 1.015 (0.634,1.625) |
| **Non-Hispanic Black :   20-39th percentile** | 1.043 (0.713,1.526) |
| **Non-Hispanic Multiple Races :   20-39th percentile** | 1.169 (0.518,2.638) |
| **Other/Unknown :   20-39th percentile** | 0.914 (0.707,1.18) |
| **Hispanic Non-White :   40-59th percentile** | 1.111 (0.479,2.58) |
| **Hispanic White :   40-59th percentile** | 1.094 (0.756,1.583) |
| **Non-Hispanic Asian :   40-59th percentile** | 0.917 (0.516,1.63) |
| **Non-Hispanic Black :   40-59th percentile** | 1.073 (0.729,1.579) |
| **Non-Hispanic Multiple Races :   40-59th percentile** | 1.248 (0.559,2.783) |
| **Other/Unknown :   40-59th percentile** | 0.87 (0.647,1.17) |
| **Hispanic Non-White :   60-79th percentile** | 1.308 (0.538,3.183) |
| **Hispanic White :   60-79th percentile** | 0.881 (0.585,1.326) |
| **Non-Hispanic Asian :   60-79th percentile** | 0.944 (0.479,1.861) |
| **Non-Hispanic Black :   60-79th percentile** | 1.172 (0.807,1.701) |
| **Non-Hispanic Multiple Races :   60-79th percentile** | 1.614 (0.701,3.717) |
| **Other/Unknown :   60-79th percentile** | 1.028 (0.746,1.416) |
| **Hispanic Non-White :   80-100th percentile** | 2.105 (0.894,4.956) |
| **Hispanic White :   80-100th percentile** | 1.159 (0.735,1.828) |
| **Non-Hispanic Asian :   80-100th percentile** | 0.426 (0.128,1.417) |
| **Non-Hispanic Black :   80-100th percentile** | 1.008 (0.682,1.492) |
| **Non-Hispanic Multiple Races :   80-100th percentile** | 1.108 (0.419,2.927) |
| **Other/Unknown :   80-100th percentile** | 1.114 (0.78,1.591) |

**Supplemental Table 14.** Multivariable Logistic regression models evaluating covariates associated with neurological manifestations known to be associated with PASC among a) SARS-CoV-2 positive cohort, b) SARS-CoV-2 negative cohort and c) SARS-CoV-2 acute respiratory infection cohort.

| **Variable Label** | **Odds Ratio Positive Cohort (2.5%, 97.5%)** | **Odds Ratio Negative Cohort (2.5%, 97.5%)** | **Odds Ratio Respiratory Cohort (2.5%, 97.5%)** |
| --- | --- | --- | --- |
| **Age Group (REF = 6-11)** | | | |
| **<5** | **0.509 (0.443,0.584)** | **0.594 (0.548,0.643)** | **0.547 (0.462,0.648)** |
| **12-17** | **1.638 (1.461,1.837)** | **1.825 (1.704,1.954)** | **1.913 (1.635,2.239)** |
| **18-20** | **1.484 (1.27,1.736)** | **1.41 (1.272,1.564)** | **1.357 (1.04,1.77)** |
| **Race/Ethnicity (REF = Non-Hispanic White)** | | | |
| **Hispanic Non-White** | 0.939 (0.695,1.268) | 0.856 (0.704,1.04) | 0.792 (0.501,1.254) |
| **Hispanic White** | **0.711 (0.605,0.837)** | **0.73 (0.656,0.813)** | **0.624 (0.488,0.797)** |
| **Non-Hispanic Asian** | **0.524 (0.382,0.718)** | **0.685 (0.592,0.794)** | **0.668 (0.464,0.962)** |
| **Non-Hispanic Black** | **0.621 (0.537,0.719)** | **0.613 (0.558,0.673)** | **0.526 (0.423,0.655)** |
| **Non-Hispanic Multiple Races** | **0.924 (0.69,1.238)** | **0.938 (0.801,1.097)** | **0.691 (0.465,1.026)** |
| **Other/Unknown** | **0.8 (0.704,0.909)** | **0.712 (0.657,0.77)** | **0.794 (0.668,0.943)** |
| **ADI (REF = 0-19th Percentile)** | | | |
| **20-39th percentile** | 1.046 (0.921,1.19) | 1.029 (0.957,1.107) | 1.119 (0.935,1.338) |
| **40-59th percentile** | **1.228 (1.073,1.406)** | **1.149 (1.06,1.244)** | **1.324 (1.098,1.596)** |
| **60-79th percentile** | 1.122 (0.968,1.302) | **1.216 (1.114,1.328)** | **1.497 (1.227,1.827)** |
| **80-100th percentile** | 1.067 (0.906,1.256) | 1.104 (0.997,1.223) | **1.294 (1.029,1.627)** |
| **Period of Index Event (REF = Mar-May 2020)** | | | |
| **Jun-Aug 2020** | 1.009 (0.653,1.558) | 0.974 (0.823,1.152) | 1.053 (0.645,1.718) |
| **Sep-Nov 2020** | 1.253 (0.848,1.853) | 0.966 (0.819,1.139) | 1.059 (0.676,1.661) |
| **Dec-Feb 2021** | 1.027 (0.704,1.499) | 1.093 (0.927,1.288) | 1.209 (0.768,1.902) |
| **Mar-May 2021** | 1.032 (0.693,1.535) | 1.055 (0.895,1.244) | 0.88 (0.557,1.39) |
| **Jun-Aug 2021** | 1.383 (0.932,2.053) | 1.048 (0.886,1.238) | 1.109 (0.71,1.733) |
| **Sep-Nov 2021** | **1.555 (1.068,2.265)** | **1.206 (1.027,1.415)** | 1.383 (0.905,2.113) |
| **Dec-Feb 2022** | **1.527 (1.062,2.196)** | **1.212 (1.029,1.428)** | 1.535 (0.999,2.357) |
| **Mar-Jul 2022** | **0.519 (0.34,0.791)** | **0.567 (0.469,0.684)** | **0.499 (0.312,0.8)** |
| **PMCA - Respiratory (REF = Non-Chronic)** | | | |
| **Chronic** | **1.804 (1.401,2.323)** | **1.984 (1.731,2.273)** | **2.191 (1.589,3.019)** |
| **Complex Chronic** | **2.009 (1.75,2.307)** | **1.734 (1.606,1.872)** | **2.457 (2.018,2.991)** |

**Supplemental Table 15**. Multivariable Logistic regression models evaluating covariates associated with neurologic PASC stratified by a) non severe acute SARS-CoV-2 infection and b) severe acute SARS-CoV-2 infection.

1. Non-severe

| **Variable Label** | **Odds Ratio (2.5%, 97.5%)** |
| --- | --- |
| **Age Group (REF = 6-11)** | |
| **- <5** | **0.462 (0.375,0.57)** |
| **- 12-17** | **1.479 (1.257,1.742)** |
| **- 18-20** | **1.402 (1.136,1.731)** |
| **Race/Ethnicity (REF = Non-Hispanic White)** | |
| **- Hispanic Non-White** | 0.992 (0.674,1.46) |
| **- Hispanic White** | **0.553 (0.442,0.692)** |
| **- Non-Hispanic Asian** | **0.509 (0.337,0.768)** |
| **- Non-Hispanic Black** | **0.579 (0.469,0.715)** |
| **- Non-Hispanic Multiple Races** | 0.686 (0.424,1.11) |
| **- Other/Unknown** | **0.756 (0.635,0.901)** |
| **ADI (REF( = 0-19th Percentile)** | |
| **- 20-39th percentile** | 1.109 (0.931,1.321) |
| **- 40-59th percentile** | 1.169 (0.966,1.415) |
| **- 60-79th percentile** | 1.064 (0.862,1.315) |
| **- 80-100th percentile** | 1.039 (0.825,1.308) |
| **Period of Index Event (REF = Mar-May 2020)** | |
| **- 1 - Jun-Aug 2020** | 0.931 (0.504,1.722) |
| **- 2 - Sep-Nov 2020** | 1.221 (0.693,2.151) |
| **- 3 - Dec-Feb 2021** | 0.986 (0.569,1.707) |
| **- 4 - Mar-May 2021** | 1.052 (0.59,1.874) |
| **- 5 - Jun-Aug 2021** | 1.6 (0.907,2.823) |
| **- 6 - Sep-Nov 2021** | 1.552 (0.895,2.69) |
| **- 7 - Dec-Feb 2022** | 1.53 (0.899,2.604) |
| **- 8 - Mar-Jul 2022** | 0.535 (0.288,0.994) |
| **PMCA - Neurological (REF = Non-Chronic)** | |
| **- Chronic** | **1.583 (1.051,2.384)** |
| **- Complex Chronic** | **2.187 (1.71,2.797)** |

1. Severe

| **Label** | **Odds Ratio (2.5%, 97.5%)** |
| --- | --- |
| **Age Group (REF = 6-11)** | |
| **- <5** | **0.539 (0.447, 0.649)** |
| **- 12-17** | **1.763 (1.5, 2.073)** |
| **- 18-20** | **1.492 (1.176, 1.893)** |
| **Race/Ethnicity (REF = Non-Hispanic White)lab** | |
| **- Hispanic Non-White** | 0.813 (0.503, 1.314) |
| **- Hispanic White** | 0.986 (0.777, 1.252) |
| **- Non-Hispanic Asian** | **0.515 (0.313, 0.846)** |
| **- Non-Hispanic Black** | **0.652 (0.533, 0.799)** |
| **- Non-Hispanic Multiple Races** | 1.129 (0.779, 1.637) |
| **- Other/Unknown** | **0.82 (0.679, 0.989)** |
| **ADI^a^ (REF = 0-19th Percentile)** | |
| **- 20-39th percentile** | 0.987 (0.817, 1.192) |
| **- 40-59th percentile** | **1.303 (1.074, 1.581)** |
| **- 60-79th percentile** | 1.204 (0.975, 1.486) |
| **- 80-100th percentile** | 1.117 (0.884, 1.412) |
| **Period of Index Event (REF = Mar-May 2020)** | |
| **- Jun-Aug 2020** | 1.143 (0.612, 2.134) |
| **- Sep-Nov 2020** | 1.3 (0.755, 2.237) |
| **- Dec-Feb 2021** | 1.101 (0.652, 1.859) |
| **- Mar-May 2021** | 1.035 (0.597, 1.795) |
| **- Jun-Aug 2021** | 1.186 (0.68, 2.067) |
| **- Sep-Nov 2021** | 1.601 (0.955, 2.684) |
| **- Dec-Feb 2022** | 1.593 (0.966, 2.625) |
| **- Mar-Jul 2022** | 0.532 (0.299, 0.948) |
| **PMCA - Neurological (REF = Non-Chronic)** | |
| **- Chronic** | **1.968 (1.424,2.719)** |
| **- Complex Chronic** | **1.986 (1.676,2.353)** |

^a^ADI- Area Deprivation Index (ADI) is a measure of social vulnerability that ranks census block groups by socioeconomic disadvantage in an area of interest and is calculated using factors such as income, education, employment, and housing quality. A block group with a ranking of 1 indicates the lowest level of “disadvantage” within the nation and an ADI with a ranking of 100 indicates the highest level of “disadvantage”.

**Supplemental Table 16**. Neurologic PASC models stratified by presence of an underlying neurologic condition

| **Variable Label** | **Non-Chronic Odds Ratio** | **Chronic Odds Ratio** | **Complex Chronic Odds Ratio** |
| --- | --- | --- | --- |
| **Race/Ethnicity - Hispanic Non-White** | 1.168 (0.826,1.652) | 0.345 (0.084,1.419) | 0.748 (0.385,1.453) |
| **Race/Ethnicity - Hispanic White** | **0.68 (0.558,0.828)** | 0.869 (0.53,1.424) | 0.795 (0.557,1.136) |
| **Race/Ethnicity - Non-Hispanic Asian** | **0.462 (0.305,0.698)** | 0.714 (0.256,1.994) | 0.626 (0.357,1.1) |
| **Race/Ethnicity - Non-Hispanic Black** | **0.623 (0.518,0.75)** | **0.536 (0.35,0.82)** | **0.661 (0.497,0.88)** |
| **Race/Ethnicity - Non-Hispanic Multiple Races** | 0.747 (0.493,1.133) | 0.445 (0.137,1.44) | 1.443 (0.911,2.285) |
| **Race/Ethnicity - Other/Unknown** | 0.868 (0.744,1.013) | 0.854 (0.565,1.291) | **0.648 (0.493,0.85)** |

**Supplemental Figure 2.** Forest plot of Respiratory PASC models demonstrating odds ratios and 95% confidence intervals for Social Vulnerability Index subthemes


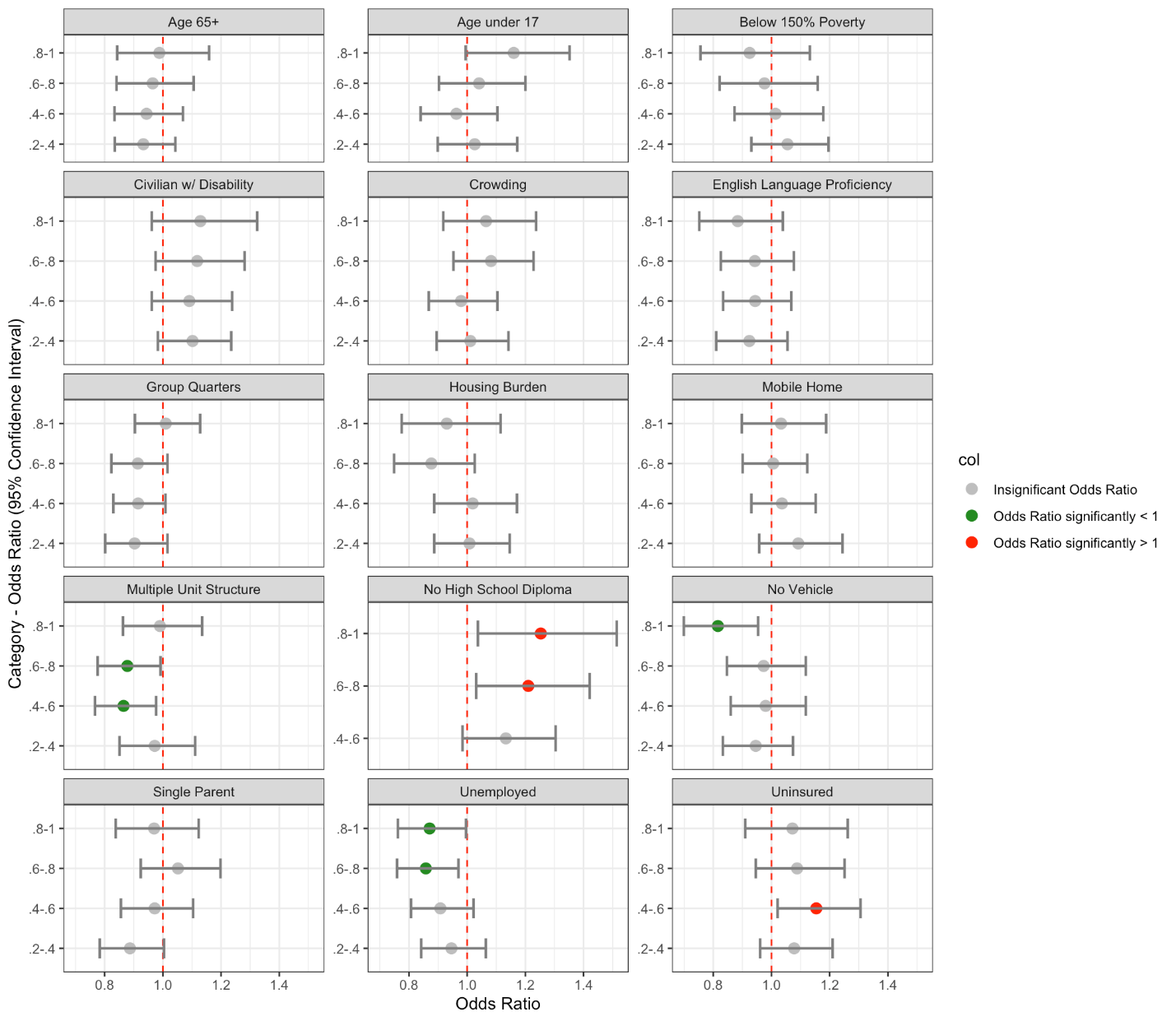


Forest plot showing models for respiratory PASC with odds ratios for as dots with lines representing 95% confidence intervals of different social vulnerability index subtheme percentiles. Red dots represent significant odds ratios > 1, green dots represent significant odds ratios < 1, grey dots represent insignificant odds ratios.

**Supplemental Figure 3.** Forest plot of Neurologic PASC models demonstrating odds ratios and 95% confidence intervals for Social Vulnerability Index subthemes


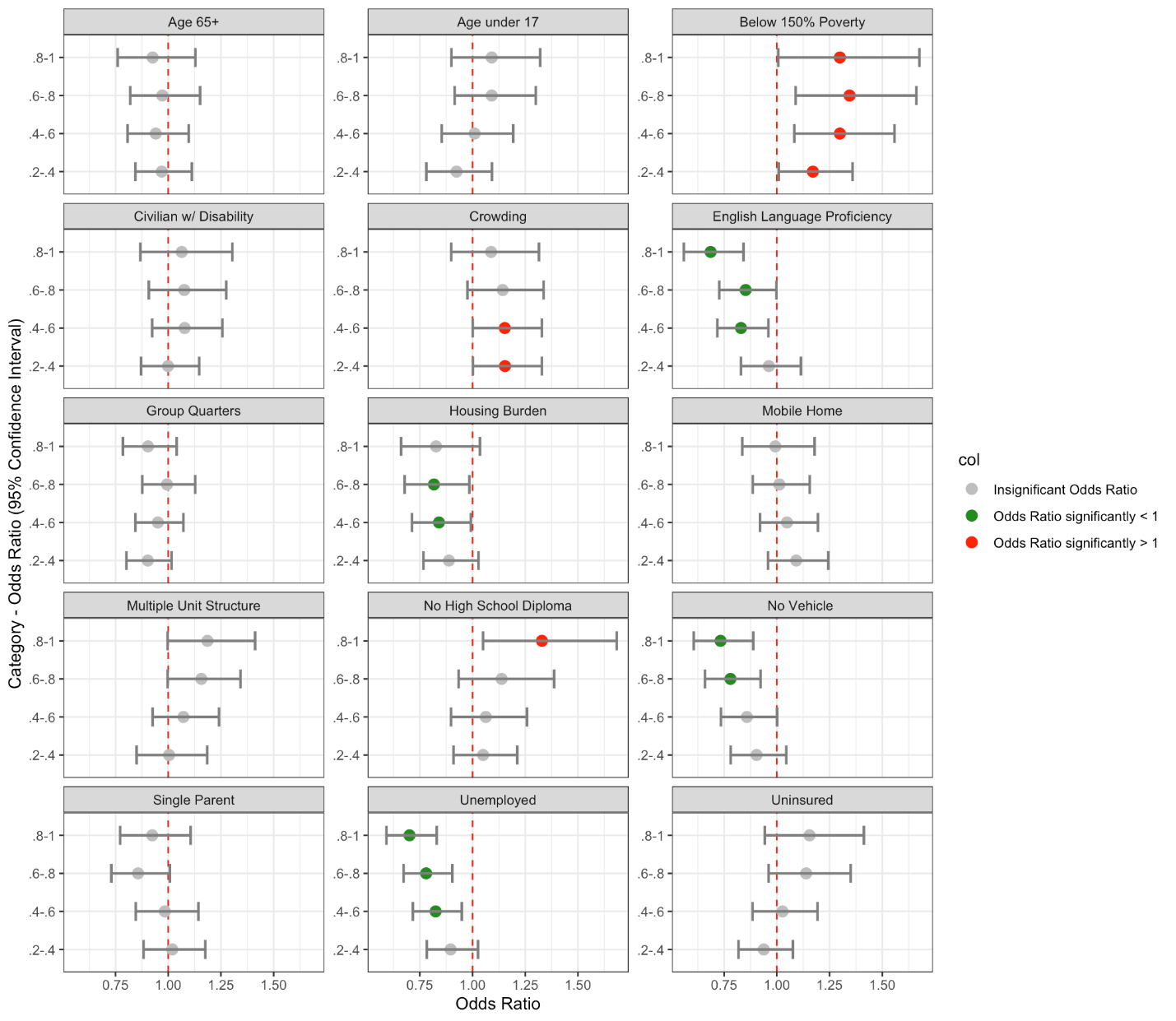


Forest plot showing models for neurologic PASC with odds ratios for as dots with lines representing 95% confidence intervals of different social vulnerability index subtheme percentiles. Red dots represent significant odds ratios > 1, green dots represent significant odds ratios < 1, grey dots represent insignificant odds ratios.
